# CHAtRF Modulates Cardiac Hypertrophy via SRSF5-Dependent Regulation of Psmg4 Alternative Splicing

**DOI:** 10.34133/research.1202

**Published:** 2026-03-26

**Authors:** Lu-Yu Zhou, Kai Wang, Ying-Hui Li, Shao-Cong Wang, Xin-Zhe Chen, Cui-Yun Liu, Xin-Min Li, Yu-Qin Wang, Shu-Fang Cai, Su-Min Yang, Yun-Hong Wang, Fang Liu, Kun Wang

**Affiliations:** ^1^ Department of Cardiovascular Surgery, Institute of Chronic Diseases, The Affiliated Hospital of Qingdao University, Qingdao 266021, China.; ^2^ Department of Medical Laboratory, Central China Subcenter of National Center for Cardiovascular Diseases, Henan Cardiovascular Disease Center, Fuwai Central-China Cardiovascular Hospital, Central China Fuwai Hospital of Zhengzhou University, Zhengzhou 450046, China.; ^3^ Hypertension Center, Beijing Anzhen Hospital, Capital Medical University, Beijing 100029, China.; ^4^ Guangxi Key Laboratory of Diabetic Systems Medicine, and Department of Anatomy, Guilin Medical University, Guilin 541199, China.

## Abstract

tRNA-derived small RNAs (tsRNAs) or tRNA-derived fragments (tRFs) are an important class of regulatory molecules whose role in cardiac hypertrophy remains largely unknown. Here, we identified a novel tRF contributing to the regulation of cardiac hypertrophy that we termed CHAtRF (cardiac hypertrophy-associated tRF). The CHAtRF level was increased in mice and in patients with cardiac hypertrophy. CHAtRF deficiency attenuated angiotensin II (AngII)-induced cardiac hypertrophy and restored the heart function, while CHAtRF overexpression enhanced hypertrophic responses. Mechanistically, CHAtRF directly interacts with SRSF5 and blocks SRSF5 to bind with Psmg4 pre-mRNA, which mediates alternative splicing of Psmg4 pre-mRNA and promotes exon 2 skipping of Psmg4. CHAtRF-dependent alternative splicing of Psmg4 inhibits the expression of Psmg4 full-length isoform, resulting in progression of pathological hypertrophy. The ability of CHAtRF to regulate hypertrophy was confirmed in hiPSC-CMs, and CHAtRF serum levels are higher in individuals with myocardial hypertrophy or heart failure. Our findings reveal new insights into the previously unrecognized role of tsRNAs during cardiac hypertrophy, which provide potential novel therapeutic targets for pathological hypertrophy and might serve as potential biomarkers for diagnosing cardiac hypertrophy and heart failure.

## Introduction

Heart failure (HF) represents a multifaceted clinical syndrome arising from structural or functional abnormalities that compromise ventricular filling or blood ejection. As a leading contributor to global morbidity and mortality, HF frequently results in marked decreases in life expectancy [1–3]. Myocardial hypertrophy, defined by pathological enlargement of cardiomyocytes and ventricular wall thickening, constitutes a crucial compensatory mechanism in response to sustained hemodynamic stress [4,5]. Persistent cardiac hypertrophy ultimately leads to arrhythmia, HF, and death [6,7]. Therapeutic strategies targeting the suppression of pathological hypertrophy and maladaptive cardiac remodeling show promise for HF management. Nevertheless, the precise molecular mechanisms governing cardiac hypertrophy development require further investigation.

tRNA-derived small RNAs (tsRNAs) or tRNA-derived fragments (tRFs) are a novel class of noncoding RNAs produced by cleavage of mature tRNA or precursor tRNA (pre-tRNA) by endonuclease under stress conditions [8]. According to the different cleavage sites on the parental tRNA, tsRNAs can be divided into tRF-1, tRF-2, tRF-3, tRF-5, i-tRF, 5′tiRNA, and 3′tiRNA isoforms [9,10]. tsRNAs can regulate gene expression through miRNA-like functions. In addition, as a class of regulatory molecules with multiple functions, it is involved in protein translation, intercellular communication, immune response, and cell apoptosis [11,12]. tsRNAs have been shown to be involved in the progression of various cardiovascular diseases (CVDs) [13]. tsRNA-5008a induces ferroptosis in cardiomyocytes, leading to atrial structural remodeling [14]. Extracellular vesicles containing tsRNAs can promote aortic valve calcification by regulating mitophagy [15]. In addition, Luo et al. [16] found that tsRNAs were also involved in the mechanism of age-related atrial fibrosis. However, the role and specific mechanism of tsRNAs in cardiac hypertrophy remains unclear.

Precursor messenger RNA (pre-mRNA) requires a series of special splicing reactions to form mature mRNA. Alternative splicing (AS) of genes has a strict regulatory network and plays an important physiological role during development. AS of pre-mRNA is closely related to CVD. Serine/arginine-rich (SR) splicing factors (SRSFs) mainly located in the nuclear speckles, including SRSF1 to SRSF12, play a crucial role in the regulation of pre-mRNA AS [17,18]. SRSF1 to SRSF7 and SRSF10 have been reported to shuttle between the nucleus and cytoplasm, which endows SRSFs with additional functions such as nuclear cytoplasmic transport and cytoplasmic function [19,20]. There is increasing evidence that SRSFs are involved in the progression of CVD and play important roles [21,22]. SRSF5 consists of 2 N-terminal RNA recognition motifs (RRMs) and an arginine/serine-rich (RS) domain [23]. It has been reported that SRSF5 promotes AS of Myom1 to maintain normal cardiac development, and SRSF5-deficient mice are peripartum lethal [24]. However, the function and mechanism of SRSF5 in pathological cardiac hypertrophy remain largely unknown.

Proteasome assembly chaperone 4 (PSMG4 or PAC4) is a chaperone protein that plays a crucial role in promoting the correct assembly of the 20S proteasome and can affect protein degradation [25]. PSMG4 works synergistically with other PACs to stabilize intermediate complexes. Disruption of PSMG4 leads to incomplete proteasome assembly, resulting in accumulation of misfolded proteins and cellular stress [26]. Recent studies highlight PSMG4’s role in stem cell regulation and targeted therapies [27,28]. Yet, the role and molecular basis of PSMG4 in CVDs remain unclear. Function and regulation of alternative PSMG4 pre-mRNA splicing in cardiac hypertrophy have not been reported thus far.

Here, we found that the cardiac hypertrophy-associated tRF (CHAtRF) level is increased in mice and in the myocardium of patients with cardiac hypertrophy. CHAtRF deficiency attenuated angiotensin II (AngII)-induced cardiac hypertrophy and restored the heart function, while CHAtRF overexpression enhanced hypertrophic responses. In addition, we uncovered that SRSF5 functions as an AS regulator of Psmg4 pre-mRNA. CHAtRF directly interacts with SRSF5 and blocks SRSF5 to bind with Psmg4 pre-mRNA, promoting exon 2 skipping of Psmg4 pre-mRNA, and thereby decreasing the relative levels of full-length (FL) Psmg4 and increasing Psmg4-S abundance. CHAtRF-mediated reduction of Psmg4 full-length isoform results in progression of pathological cardiac hypertrophy, suggesting a critical role for the CHAtRF–SRSF5–Psmg4 axis as regulators of cardiac hypertrophy. Our findings provide novel insights into the role of tsRNAs in cardiac hypertrophy, and targeting the CHAtRF–SRSF5–Psmg4 axis represents a promising therapeutic strategy for treating pathological cardiac remodeling and HF.

## Results

### CHAtRF is up-regulated during cardiac hypertrophy and hypertrophic cardiomyopathy

**Fig. 1.**
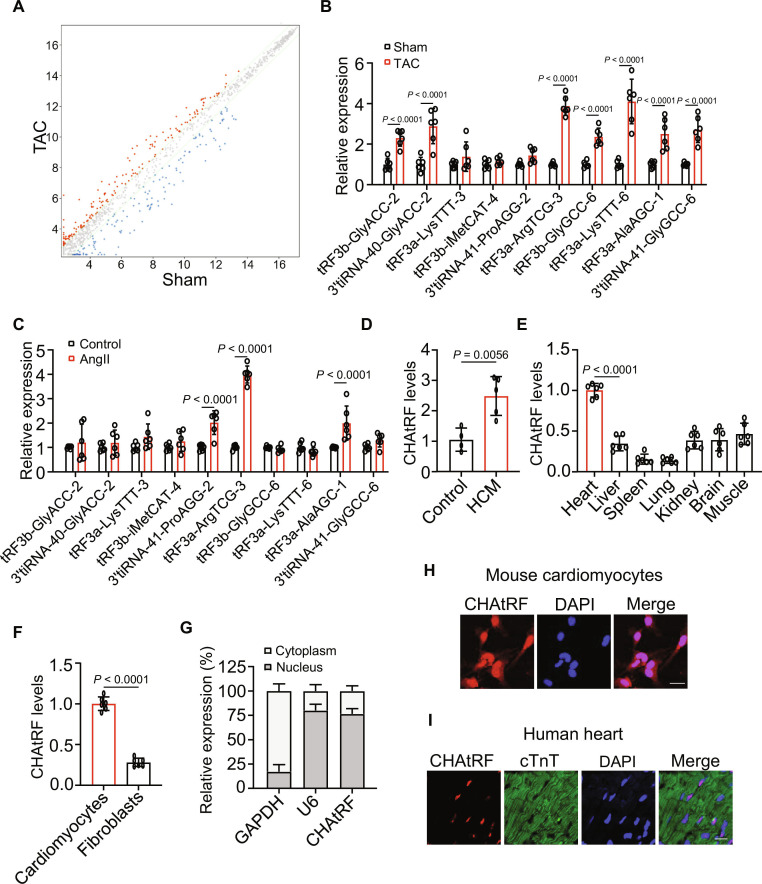
CHAtRF is up-regulated during cardiac hypertrophy and HCM. (A) Scatterplot of differential expression of tsRNAs assessed from tsRNA microarray data. Red dots denote up-regulated genes, and blue dots denote down-regulated genes. (B and C) Quantitative real-time PCR (qPCR) analysis of up-regulated tsRNAs in TAC-induced cardiac hypertrophy mouse hearts selected from tsRNA microarray data (B) or AngII-treated cardiomyocytes (C) (*n* = 6 independent experiments). (D) qPCR analysis of CHAtRF levels in the normal myocardium of control patients (*n* = 4) and the myocardium of patients with hypertrophic cardiomyopathy (HCM; *n* = 5). (E) Determination of relative expression of CHAtRF in different tissues of adult mice by qPCR (*n* = 6 mice per group). (F) Detection of relative expression of CHAtRF in cardiomyocytes and fibroblasts (*n* = 6 independent experiments). (G) The levels of CHAtRF in the cytoplasm or nuclear fractions of cardiomyocytes were determined by qPCR. U6 and GAPDH were used as internal controls (*n* = 6 independent experiments). (H and I) Representative images of fluorescence in situ hybridization with junction-specific probes of CHAtRF in the neonatal mouse cardiomyocytes (H) and the myocardial sections from healthy donors (I) demonstrate its subcellular localization. Scale bar, 20 μm. Data are presented as mean ± SD. Data presented in (B) and (C) were analyzed by 2-way ANOVA with Tukey post hoc test. Data presented in (D) and (F) were analyzed by Student,s *t* test (2-tailed). Data presented in (E) was analyzed by one-way ANOVA with Tukey post hoc test.

To evaluate the role of tsRNAs in cardiac hypertrophy, an unbiased screening was performed in a transverse aortic constriction (TAC) surgery-induced hypertrophic mouse model (Fig. 1A and B, Fig. S1A, and Table S1) and AngII-treated hypertrophic neonatal mouse cardiomyocytes (Fig. 1C and Fig. S1B) using tsRNA microarray and quantitative polymerase chain reaction with reverse transcription (RT-qPCR) analysis. Among them, the tsRNA with the greatest change in expression was tRF3a-ArgTCG-3 (Fig. S1C), and we named it CHAtRF. We then detected the expression of CHAtRF in the hearts of patients with hypertrophic cardiomyopathy HCM (Table S2), and the results showed that CHAtRF levels were increased in HCM patients compared with healthy controls (Fig. 1D). Next, we determined the CHAtRF expression profile in different organs and observed a higher CHAtRF content in the heart than in other organs (Fig. 1E). CHAtRF was remarkably higher in cardiomyocytes than in fibroblasts (Fig. 1F), and was mainly distributed in the nuclei of mouse and human cardiomyocytes (Fig. 1G to I). Taken together, these data suggest that CHAtRF is up-regulated under hypertrophic conditions and may be involved in cardiac hypertrophy and HF.

### CHAtRF deficiency attenuates pathological cardiac hypertrophy

**Fig. 2.**
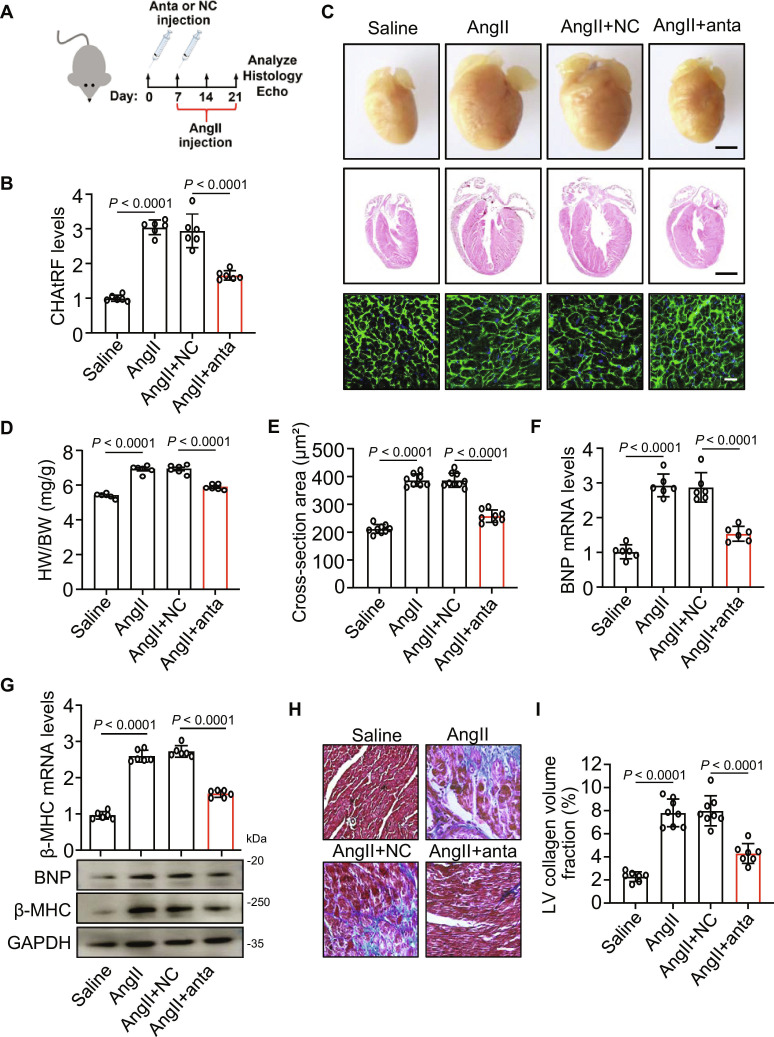
CHAtRF deficiency attenuates pathological cardiac hypertrophy. (A to G) Mice were transfected with CHAtRF anta or its negative control (NC), and then they were injected with AngII. (A) Schematic diagram of the injection of CHAtRF anta and the experimental procedure. Echo, echocardiography. (B) qPCR analysis of the expression level of CHAtRF (*n* = 6 mice per group). (C) Representative images of gross morphology of hearts (upper row). Scale bar, 2 mm. Representative images of coronal sections of heart stained with hematoxylin and eosin (middle row). Scale bar, 2 mm. Representative images of left ventricular muscle sections stained with WGA (bottom row). Scale bar, 25 μm. (D) Heart weight (HW)/body weight (BW) ratio (*n* = 6 to 8 mice per group). (E) Analysis of the cardiomyocyte sizes in histological sections stained with WGA (*n* = 8 to 9 mice per group). (F) qPCR analysis of the expression level of BNP mRNA (*n* = 6 mice per group). (G) qPCR analysis of the expression level of β-MHC mRNA (upper panel; *n* = 6 mice per group). Immunoblotting for BNP and β-MHC protein expression (lower panel). (H) Representative images of Masson,s trichrome-stained histological sections. Scale bar, 20 μm. (I) Quantitative analysis of collagen contents in left ventricle samples (*n* = 7 to 8 mice per group). Data are presented as mean ± SD. One-way ANOVA test (B, D to G, and I).

To evaluate the functional relevance of CHAtRF in pathological cardiac hypertrophy in vivo, antagomir (anta) was delivered into the tail veins of mice, followed by continuous AngII infusion for 2 weeks (Fig. 2A). The efficiency of CHAtRF deficiency in mouse myocardial tissue was verified by RT-qPCR (Fig. S2A and Fig. 2B). CHAtRF deficiency mice showed significantly decreased heart sizes (Fig. 2C), heart weight/body weight ratios (Fig. 2D), heart weight/tibia length ratio (Fig. S2B), and cross-sectional area of cardiomyocytes (Fig. 2E) compared to negative control (NC)-treated mice after AngII infusion. The expression of hypertrophic genes (Fig. 2F and G and Fig. S2C) was decreased in CHAtRF deficiency mice upon hypertrophic stimuli. Moreover, AngII-mediated increases in cardiac fibrosis were significantly reduced in CHAtRF deficiency mice (Fig. 2H and I). Compared to NC mice, CHAtRF-deficient mice exhibited improved cardiac function upon AngII administration, characterized by elevated left ventricular ejection fraction (EF) and fractional shortening (FS), along with an increased mitral ratio of peak early to late diastolic filling velocity (E/A) (Fig. S2D to G).

To further investigate CHAtRF’s role in cardiac hypertrophy, we subjected CHAtRF-deficient mice (anta) and NC-treated mice to TAC. Following TAC surgery, CHAtRF-deficient mice exhibited significantly smaller heart sizes (Fig. S3A) and reduced heart weight/body weight ratios (Fig. S3B) compared to NC-treated mice. Wheat germ agglutinin (WGA) staining revealed smaller cardiomyocyte cross-sectional areas in CHAtRF-deficient mice post-TAC (Fig. S3C). Moreover, the expression of hypertrophic genes were down-regulated in CHAtRF-deficient mice under hypertrophic stimuli (Fig. S3D and E). Collectively, these findings indicate that CHAtRF deficiency prevents the development of pathological cardiac hypertrophy.

### CHAtRF regulates AngII-induced hypertrophy in cardiomyocytes

**Fig. 3.**
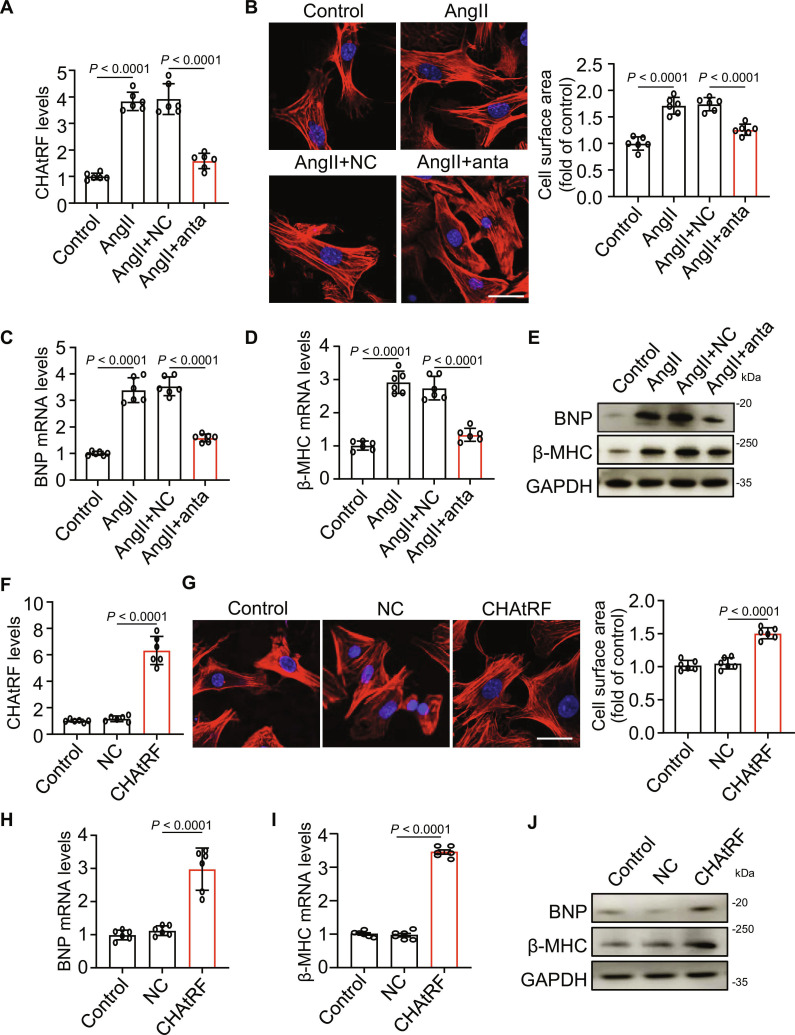
CHAtRF regulates AngII-induced hypertrophy in cardiomyocytes. (A to E) Cardiomyocytes were transfected with anta and NC for 24 h, and then cells were treated with AngII for 48 h. (A) qPCR analysis of the expression level of CHAtRF (*n* = 6 independent experiments). (B) Cardiomyocytes were stained by phalloidin (left), and quantitative analysis of the cell surface area (right) was assessed (*n* = 6 independent experiments). Scale bar, 25 μm. (C and D) Expression levels of hypertrophy genes in cardiomyocytes. qPCR results showing BNP mRNA level (C, *n* = 6 independent experiments) and β-MHC mRNA level (D, *n* = 6 independent experiments). (E) Immunoblotting for BNP and β-MHC protein expression. (F to J) Cardiomyocytes were transfected with CHAtRF agomir (CHAtRF) or its NC for 24 h. (F) qPCR analysis of the expression level of CHAtRF (*n* = 6 independent experiments). (G) Cardiomyocytes were stained by phalloidin (left), and quantitative analysis of the cell surface area (right) was assessed (*n* = 6 independent experiments). Scale bar, 25 μm. (H and I) Expression levels of hypertrophy genes in cardiomyocytes. qPCR results showing BNP mRNA level (H) and β-MHC mRNA level (I) (*n* = 6 independent experiments). (J) Immunoblotting for BNP and β-MHC protein expression. Data are presented as mean ± SD. Data presented in (A) to (D) and (F) to (I) were analyzed by one-way ANOVA with Tukey post hoc test.

We next investigated the impacts of CHAtRF on hypertrophy induced by AngII in cardiomyocytes. CHAtRF knockdown significantly attenuated the AngII-induced increase in both CHAtRF expression (Fig. S3F and Fig. 3A) and cardiomyocyte size (Fig. 3B). The expression of hypertrophic marker genes B-type natriuretic peptide (BNP) and β-myosin heavy chain (β-MHC) upon AngII treatment was suppressed by CHAtRF knockdown (Fig. 3C to E). In addition, overexpression of CHAtRF (Fig. 3F) induced hypertrophic responses, as indicated by increased cardiomyocyte size (Fig. 3G) and enforced expression levels of hypertrophic marker genes (Fig. 3H to J). Together, these results indicate that CHAtRF knockdown attenuates AngII-induced cardiomyocyte hypertrophy in vitro.

### CHAtRF binds to splicing factor SRSF5 to regulate mRNA AS

**Fig. 4.**
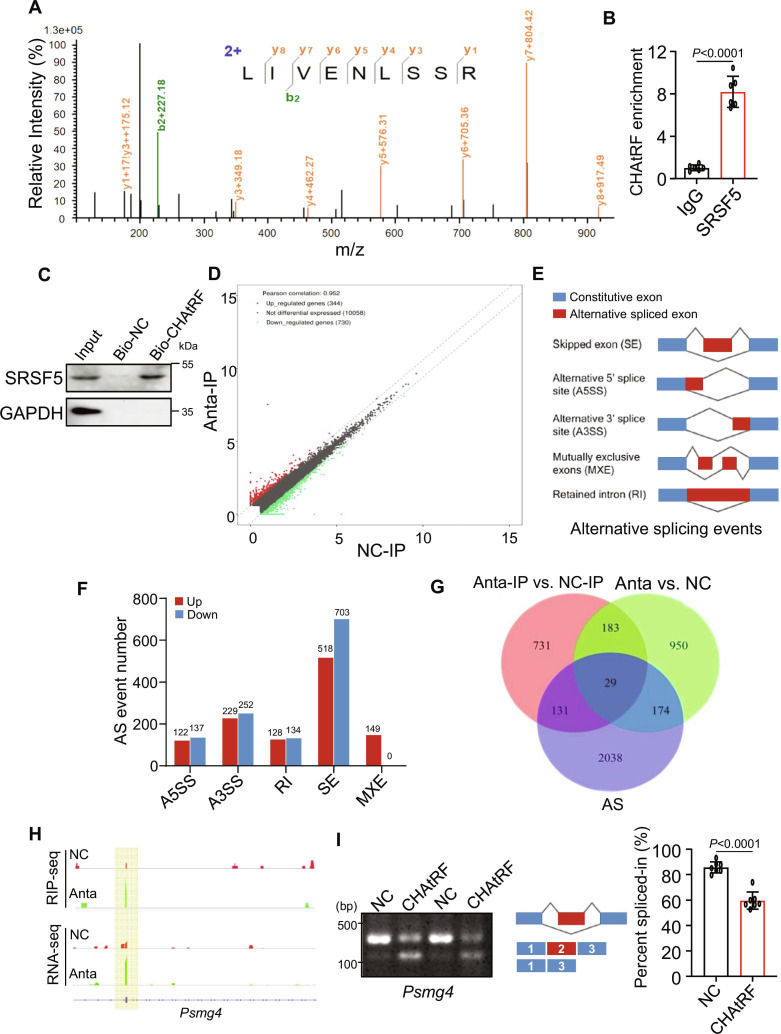
CHAtRF binds to splicing factor SRSF5 to regulate mRNA alternative splicing. (A) LC-MS/MS identification of SRSF5. (B) RNA immunoprecipitation using SRSF5 antibody followed by qPCR analysis showing CHAtRF enriched in SRSF5 fraction (*n* = 6 independent experiments). (C) RNA pull-down assay was carried out using biotinylated CHAtRF (Bio-CHAtRF) or NC (Bio-NC) and WB analysis showing that CHAtRF binds with SRSF5 protein. (D) Data of RNA immunoprecipitation sequencing with anti-SRSF5 antibody in NC and CHAtRF knockdown cells. (E) Summary of differential splicing analysis performed using anta- or NC-transfected cardiomyocytes. (F) Numbers of predicted alternative splicing (AS) events in each category upon CHAtRF deletion. (G) Venn diagrams showing overlap of RIP-seq genes, RNA-seq genes, and AS genes in CHAtRF anta- or NC-transfected cardiomyocytes. (H) Alternative sites in Psmg4 gene bound directly by SRSF5 from RIP-seq and RNA-seq using IGV software. (I) RT-PCR analysis for AS event of Psmg4 gene in NC- and CHAtRF-overexpressed cardiomyocytes. The middle panels represent the schematic diagram of indicated AS exons. Right panels show the quantification of percent spliced-in (PSI) (*n* = 7 independent experiments). Data are presented as mean ± SD. Data presented in (B) and (I) were analyzed by Student,s *t* test (2-tailed).

To decipher the underlying molecular mechanisms of how CHAtRF regulates cardiac hypertrophy, we performed biotin–streptavidin pull-down assay using biotinylated CHAtRF followed by mass spectrometry (MS) analysis to identify the potential downstream targets regulated by CHAtRF in cardiomyocytes (Fig. S4A). Among the enriched proteins, serine/arginine-rich splicing factor 5 (SRSF5), which plays a critical role during heart development [21,22], was selected for further analysis (Fig. 4A). We performed RNA immunoprecipitation (RIP) assay followed by RT-qPCR and observed the enrichment of CHAtRF in SRSF5-RNA precipitates (Fig. 4B), which suggests the interaction between CHAtRF and SRSF5 in vivo. In addition, RNA pull-down assay using biotinylated CHAtRF followed by immunoblot also confirmed the direct binding of SRSF5 and CHAtRF (Fig. 4C). Next, we further demonstrated that mRNA and protein levels of SRSF5 were not influenced by CHAtRF overexpression (Fig. S4B) or CHAtRF knockdown (Fig. S4C) in cardiomyocytes. These results suggest that CHAtRF directly binds to SRSF5 without affecting its expression in cardiomyocytes. 

Given that SRSF5 is critically involved in pre-mRNA AS and interacts with CHAtRF, which is mainly distributed in the nucleus of cardiomyocytes, we speculate that CHAtRF might be involved in the regulation of RNA AS of its target gene. To test this, we performed RIP-sequencing (RIP-seq) (Table S3) using SRSF5 antibody (Fig. 4D) and high-throughput RNA-sequencing (RNA-seq) analysis (Fig. S4D) (Table S4) in cardiomyocytes with knocked down CHAtRF. As expected, the RNA-seq analysis identified a large number of mRNA splicing changes in CHAtRF silencing cardiomyocytes, including skipped exons (SEs), alternative 5′ splice sites (A5SSs), alternative 3′ splice sites (A3SSs), mutually exclusive exons (MXEs), and retained introns (RIs) (Fig. 4E) (Table S5). Among the AS events affected by CHAtRF ablation, SE was the predominant splicing type (Fig. 4F). Upon comparing RIP-seq, RNA-seq, and AS events, we found that 29 genes had both differentially AS and expressed genes (Fig. 4G). Among them, proteasome assembly chaperone 4 (Psmg4) attracted our attention, the enrichment level of which bound to SRSF5 protein significantly increased in CHAtRF-deficient cardiomyocytes compared with that in the NC (Fig. 4H). To clarify the relationship between the CHAtRF and the AS of Psmg4, we examined Psmg4 splicing by RT-PCR using a primer set designed to amplify both the long variant transcript of Psmg4 (Psmg4-L) and the short transcript variant of Psmg4 (Psmg4-S). Our results showed that CHAtRF decreased the percent spliced-in (PSI) of Psmg4-L (Fig. 4I) and increased the exclusion of Psmg4 exon 2, suggesting that CHAtRF promotes the Psmg4 exon 2 skipping. Therefore, Psmg4 was selected to further explore its contribution to cardiac hypertrophy.

### CHAtRF blocks SRSF5 to mediate the AS of Psmg4

**Fig. 5.**
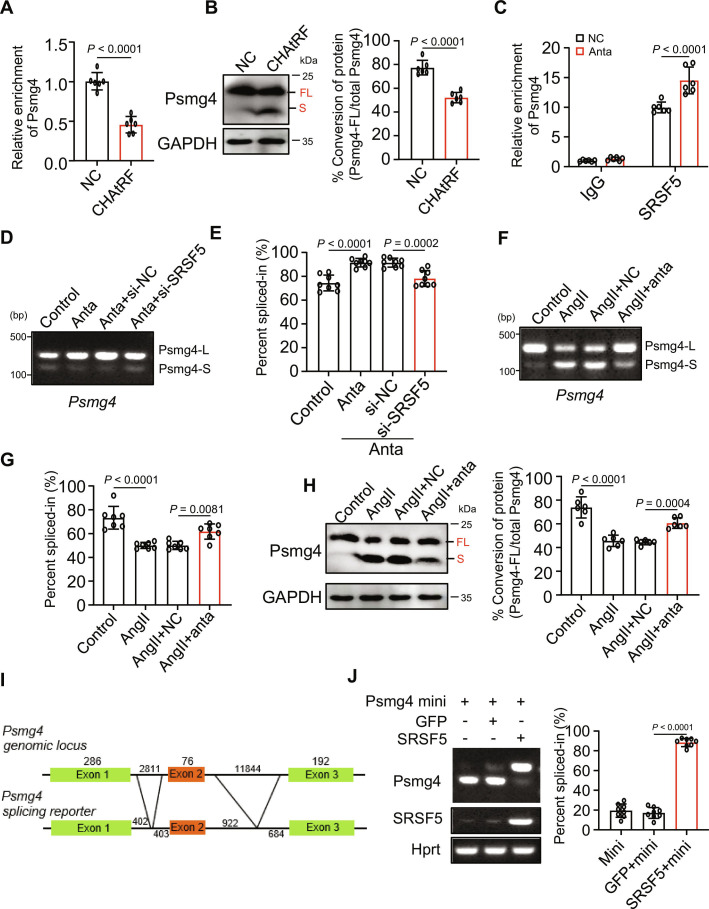
CHAtRF blocks SRSF5 to mediate the AS of Psmg4. (A) RIP-qPCR analysis showing relative binding level of SRSF5 to Psmg4 mRNA in NC or CHAtRF-overexpressed cardiomyocytes (*n* = 6 independent experiments). (B) WB analyses of the expression of Psmg4 protein in NC or CHAtRF-overexpressed cardiomyocytes. GAPDH was used as a loading control. (C) RIP-qPCR analysis showing relative binding level of SRSF5 to Psmg4 mRNA in NC- or anta-transfected cardiomyocytes (*n* = 6 independent experiments). (D) RT-PCR analysis for AS events of Psmg4 gene in cardiomyocytes transfected with anta, si-NC, or si-Srsf5. (E) Quantification of PSI (*n* = 8 independent experiments). (F) Cardiomyocytes were transfected with anta or NC for 24 h, and then cells were treated with AngII. RT-PCR analysis for AS events of Psmg4 gene. (G) Quantification of PSI (*n* = 7 independent experiments). (H) WB analyses of the expression of Psmg4 protein in cardiomyocytes transfected with anta or NC for 24 h and then treated with AngII. GAPDH was used as a loading control (*n* = 6 independent experiments). (I) Schematics for the full-length Psmg4 domains and the Psmg4 minigene-splicing reporter based on the Psmg4 genomic locus. Lengths of exons and introns are indicated. (J) 293T cells were transfected with Psmg4 mini plasmid and adenovirus harboring Srsf5. RT-PCR analysis of the splicing pattern of the Psmg4 mini reporter (left panel) and quantification of RT-PCR data (right panel) were shown (*n* = 8 independent experiments). Data are presented as mean ± SD. Data presented in (A) and (B) were analyzed by Student,s *t* test (2-tailed). Data presented in (C) were analyzed by 2-way ANOVA with Tukey post hoc test. Data presented in (E), (G), (H), and (J) were analyzed by one-way ANOVA with Tukey post hoc test.

We next wanted to examine how CHAtRF regulates the pre-mRNA AS of Psmg4. Considering the ability of CHAtRF to interact with splicing factor SRSF5, we examined the binding affinity of the Psmg4 mRNA to SRSF5 by RIP-qPCR assay in cardiomyocytes overexpressing CHAtRF. The results showed that the enrichment level of Psmg4 pre-mRNA bound to SRSF5 protein significantly decreased in CHAtRF-overexpressed cardiomyocytes compared with that in the control (Fig. 5A). As the above data had shown a significant Psmg4 mRNA AS change in CHAtRF-overexpressed cells (Fig. 4I), we thus performed Western blot (WB) assay and the result confirmed that AS-changed Psmg4 was also abnormally expressed in protein levels. Enforced expression of CHAtRF promoted the skipping of Psmg4 exon 2, leading to a shift in isoform expression characterized by an increased level of the short isoform and a corresponding decrease in the long isoform compared to the control (Fig. 5B). In addition, we observed an increased enrichment of Psmg4 pre-mRNA bound to SRSF5 protein in CHAtRF-knocked down cardiomyocytes compared with NCs (Fig. 5C). Knockdown of CHAtRF inhibited the Psmg4 exon 2 skipping and increased the PSI of Psmg4-L, which was reversed by SRSF5 knockdown (Fig. 5D and E). Our data further confirmed that knocking down CHAtRF suppressed the Psmg4 exon 2 skipping in AngII- or TAC-induced hypertrophic cardiomyocytes (Fig. 5F, G and Fig. S4E). WB results also revealed a significant change in protein expression levels of Psmg4 (Fig. 5H). Taken together, these results demonstrate that CHAtRF can block SRSF5 to bind with Psmg4 pre-mRNA and promote exon 2 skipping and AS of Psmg4 during hypertrophy.

To further confirm the ability of SRSF5 to modulate AS of Psmg4 and assess directly if Psmg4 exon 2 is alternatively spliced by SRSF5, we constructed a Psmg4 minigene-splicing reporter (exons 1 to 3) and investigated the role of SRSF5 in AS of Psmg4 in the 293T cell line using the canonical minigene-splicing reporter system based on the genomic splicing region of Psmg4 (Fig. 5I). According to the results, exogenous coexpression of SRSF5 with minigene of Psmg4 showed a significant increase transcript containing exon 2 compared with the control group (Fig. 5J). Furthermore, SRSF5 overexpression in cardiomyocytes also increased Psmg4 transcript containing exon 2 (Fig. S4F), suggesting its regulation of endogenous Psmg4 splice variants. Collectively, these results confirm that SRSF5 is able to directly regulate the AS of Psmg4.

### Psmg4 full-length isoform inhibits AngII-induced hypertrophy in cardiomyocytes

**Fig. 6.**
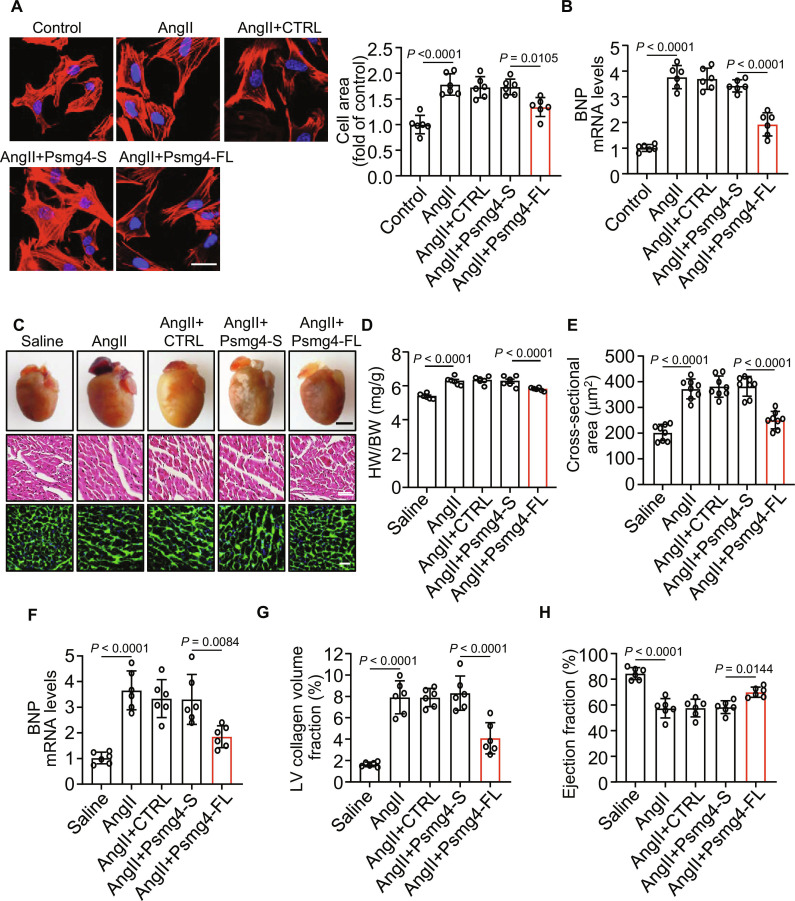
Psmg4 full-length isoform inhibits AngII-induced hypertrophy in cardiomyocytes. (A and B) Cardiomyocytes were infected with adenovirus harboring Psmg4-S, Psmg4-FL, and CTRL for 24 h, and then cells were treated with AngII for 48 h. (A) Cardiomyocytes were stained by phalloidin (left), and quantitative analysis of the cell surface area (right) was assessed (*n* = 6 independent experiments). Scale bar, 25 μm. (B) qPCR results showing BNP mRNA level (*n* = 6 independent experiments). (C to H) Adenovirus harboring Psmg4-S, Psmg4-FL, and CTRL were injected into 8- to 10-week-old mice, and AngII was treated after 1 week to induce cardiac hypertrophy. Heart samples were collected 2 weeks post-AngII treatment. (C) Top row: Representative images of gross morphology of hearts. Scale bar, 2 mm. Middle row: Result of H&E staining in heart tissues. Scale bar, 20 μm. Bottom row: Representative images of left ventricular muscle sections stained with WGA. Scale bar, 25um. (D) HW/BW ratio (*n* = 6 to 8 mice per group). (E) Analysis of the cardiomyocyte sizes in histological sections stained with WGA (*n* = 8 mice per group). (F) qPCR analysis of the expression level of BNP mRNA (*n* = 6 mice per group). (G) Quantitative analysis of collagen contents in left ventricle samples (*n* = 6 mice per group). (H) Left ventricular EF detected by echocardiography in mice (*n* = 6 mice per group). Data are presented as mean ± SD. Data presented in (A), (B), and (D) to (H) were analyzed by one-way ANOVA with Tukey post hoc test.

Next, we investigated whether Psmg4 directly regulates cardiomyocyte hypertrophy using an in vitro model of hypertrophy induced by AngII in cardiomyocytes. Given that our splicing assay showed that CHAtRF promoted the exclusion of Psmg4 exon 2, resulting in a shift of Psmg4 expression from full-length to short isoform, we thus sought to elucidate the functional impact of these 2 isoforms on the regulation of cardiomyocyte hypertrophy. Recombinant adenoviral vectors harboring full-length or short isoform were used to transfect cardiomyocytes. Results showed that AngII-induced hypertrophic effects were significantly attenuated in cardiomyocytes overexpressing the full-length isoform relative to short isoform cells based on the analysis of cardiomyocyte size (Fig. 6A) and the expression level of BNP (Fig. 6B).

In vivo, recombinant adenoviral vectors carrying the Psmg4 full-length or short isoform were intravenously administered to mice 1 week before AngII infusion (Fig. S5A). The results showed that in the Psmg4 full-length isoform group, significant decreases of heart weight/body weight ratios (Fig. 6C and D), heart weight/tibia length ratio (Fig. S5B), heart size (Fig. 6E), expression of hypertrophic gene (Fig. 6F), and myocardial fibrosis (Fig. 6G) were observed after AngII infusion relative to the Psmg4 short group. Furthermore, the full-length isoform group displayed improved cardiac function relative to the short isoform group after AngII infusion (Fig. 6H and Fig. S5C to E). Taken together, these results suggested that Psmg4 exon 2 inclusion is critical for the suppression of cardiac hypertrophy, and Psmg4 full-length isoform inhibits AngII-induced hypertrophy in cardiomyocytes and in vivo.

Our study establishes that the Psmg4 full-length isoform has anti-hypertrophic effects, but its specific molecular mechanism remains entirely unknown. We preliminarily explored its downstream mechanisms. As Psmg4 is a proteasome assembly chaperone, its functional loss might lead to misfolded protein accumulation and cellular stress [29,30]. We thus measured proteasome activity upon Psmg4 overexpression, and the results showed that Psmg4-S overexpression attenuated proteasome activity (Fig. S5F). This finding suggests that Psmg4-S overexpression may lead to misfolded protein accumulation and cellular stress. We proposed that the increase of Psmg4-S due to CHAtRF regulation may disrupt proteasome assembly or function, leading to the accumulation of misfolded proteins and subsequent activation of stress pathways that drive cardiac hypertrophy.

### Psmg4 and SRSF5 functions as downstream molecules of CHAtRF in cardiac hypertrophy

**Fig. 7.**
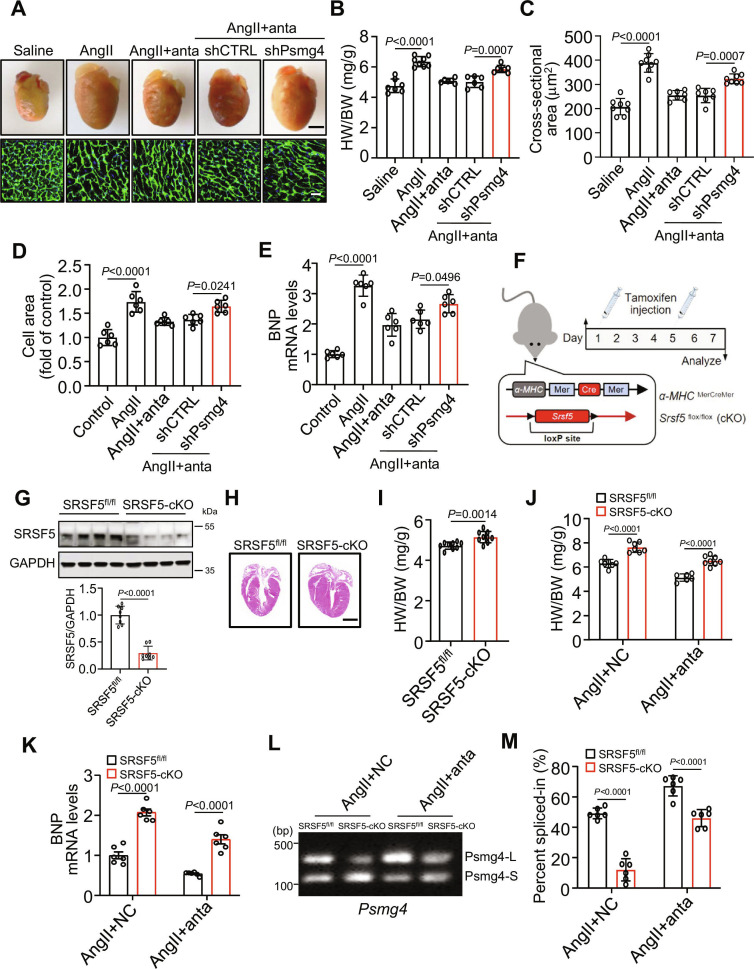
Psmg4 and SRSF5 function as downstream molecules of CHAtRF in cardiac hypertrophy. (A to C) Mice were treated with AngII and transfected with CHAtRF anta or its NC, while infected with adenovirus harboring shPsmg4 or shCTRL. (A) Top row: Representative images of gross morphology of hearts. Scale bar, 2 mm. Bottom row: Representative images of left ventricular muscle sections stained with WGA. Scale bar, 25 μm. (B) HW/BW ratio (*n* = 6 to 8 mice per group). (C) Analysis of the cardiomyocyte sizes in histological sections stained with WGA (*n* = 7 to 8 mice per group). (D and E) Cardiomyocytes were treated with AngII and transfected with CHAtRF anta or its NC, while infected with adenovirus harboring shPsmg4 or shCTRL. (D) Cardiomyocytes were stained by phalloidin, and quantitative analysis of the cell surface area was assessed (*n* = 6 independent experiments). (E) qPCR results showing BNP mRNA level (*n* = 6 independent experiments). (F) Schematic diagram of tamoxifen (TAM)-induced generation in SRSF5-cKO mice. NC mice were SRSF5*^flox/flox^* mice, and SRSF5-cKO mice were crosses between α-MHCMerCreMer mice and SRSF5*^fl/fl^* mice. (G) WB showed SRSF5 expression in SRSF5*^fl/fl^* mice and SRSF5-cKO mice (*n* = 8 mice per group). (H) Representative images of gross morphology of SRSF5-cKO mice and SRSF5*^fl/fl^* mouse hearts. Scale bar, 2 mm. (I) Analysis of HW/BW ratio in the SRSF5-cKO and SRSF5*^fl/fl^* mice (*n* = 9 mice per group). (J to M) SRSF5-cKO and SRSF5*^fl/fl^* mice were injected with AngII and transfected with CHAtRF anta or its NC. (J) HW/BW ratio (*n* = 6 to 8 mice per group). (K) qPCR results showing BNP mRNA level (*n* = 6 mice per group). (L) RT-PCR analysis of the splicing pattern of the Psmg4 gene (left panel) and quantification of RT-PCR data (right panel) (*n* = 6 independent experiments). Data are presented as mean ± SD. Data presented in (B) to (E) were analyzed by one-way ANOVA with Tukey post hoc test. Data presented in (G) to (I) were analyzed by Student,s *t* test (2-tailed). Data presented in (J), (K), and (M) were analyzed by 2-way ANOVA with Tukey post hoc test.

We assessed whether the effect of CHAtRF on hypertrophy was Psmg4-dependent. In vivo, CHAtRF inhibition blocked AngII-induced increases of heart weight/body weight ratios (Fig. 7A and B), cell size (Fig. 7C), expression of hypertrophic gene (Fig. S6A), myocardial fibrosis (Fig. S6B), and a decrease of cardiac function (Fig. S6C), while these effects were attenuated upon silencing Psmg4 (Fig. S6D and E). In cardiomyocytes, knockdown of Psmg4 also reduced the inhibitory effects of CHAtRF silencing on hypertrophy (Fig. 7D and E).

To explore the role of SRSF5 in the regulation of cardiac hypertrophy, we generated cardiac-specific SRSF5 knockout mice. Knockout of SRSF5 was generated by crossing SRSF5-floxed (SRSF5^fl/fl^) mice with Myh6-MerCreMer mice, thus allowing tamoxifen (TAM)-inducible deletion of SRSF5 [SRSF5-conditional knockout (cKO)] in cardiomyocyte (Fig. 7F). The SRSF5 level was dramatically reduced in the cardiac tissue of SRSF5-cKO mice compared with the SRSF5^fl/fl^ mice (Fig. S7A and Fig. 7G). Srsf5-cKO mice exhibited normal development to adulthood and unaffected survival rates. Under normal physiological conditions, these mice displayed only slight hypertrophic changes (Fig. 7H and I and Fig. S7B) and a decrease in cardiac function (Fig. S7C) compared to SRSF5^fl/fl^ controls. AngII-induced hypertrophic effects were significantly increased in Srsf5-cKO mice compared with SRSF5^fl/fl^ mice based on the analysis of heart weight/body weight ratios (Fig. S7D and E), cardiomyocyte size (Fig. S7F), myocardial fibrosis (Fig. S7G), the expression levels of BNP (Fig. S7H), and cardiac function (Fig. S7I). Similarly, TAC treatment also led to amplified hypertrophic effects in Srsf5-cKO mice relative to SRSF5^fl/fl^ controls (Fig. S7J).

We next sought to investigate whether CHAtRF mediated cardiac hypertrophy and the AS of Psmg4 by SRSF5. The inhibitory effects of CHAtRF silencing on AngII-induced hypertrophy were significantly reversed in Srsf5-cKO mouse heart, as indicated by increased heart weight/body weight ratios (Fig. 7J) and the expression of hypertrophic gene (Fig. 7K). Moreover, Psmg4 exon 2 inclusion induced by CHAtRF knockdown was decreased in Srsf5-cKO mouse heart (Fig. 7L). In agreement with pre-mRNA AS of Psmg4, Psmg4 full-length isoform expression was also significantly reduced in Srsf5-cKO mouse heart (Fig. 7M). We further examined the involvement of CHAtRF, SRSF5, and PSMG4 in the regulation of physiological myocardial hypertrophy, which was induced by swim training in mice [31,32]. Our results, illustrated in Fig. S8A to H, revealed no significant impact of CHAtRF, SRSF5, or PSMG4 on exercise-induced hypertrophy. This indicates that these factors do not play a regulatory role in physiological cardiac hypertrophy. Collectively, these results reveal that SRSF5 and Psmg4 are direct downstream molecules of CHAtRF in the regulation of pathological cardiac hypertrophy.

### The effects of CHAtRF knockdown in established cardiac hypertrophy

**Fig. 8.**
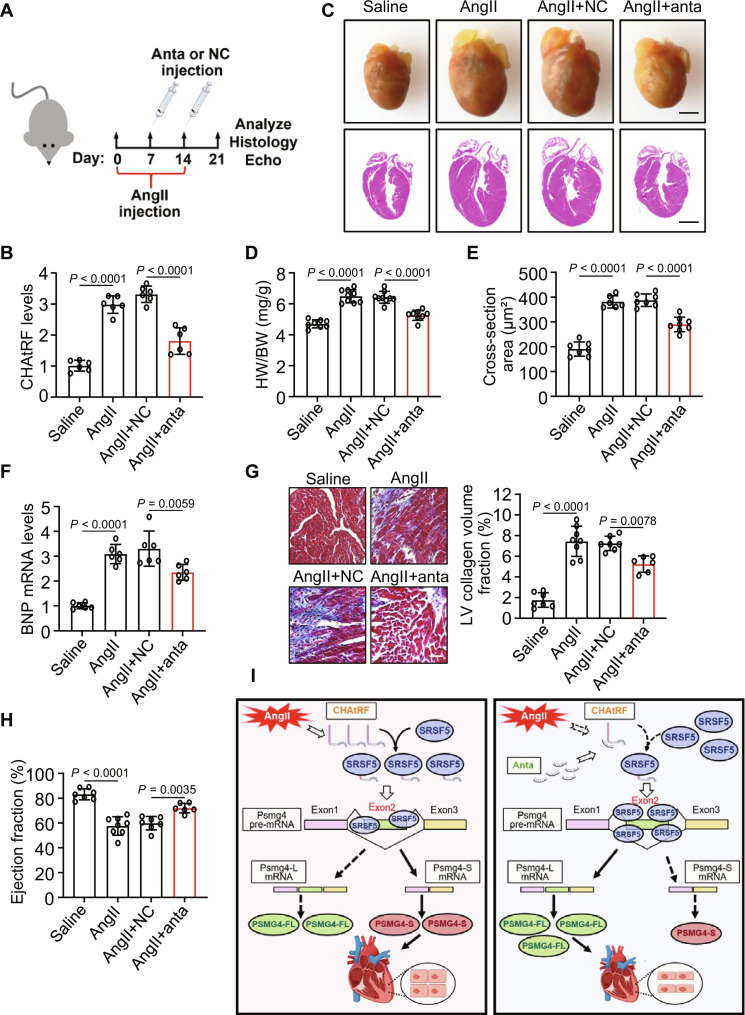
The effects of CHAtRF knockdown in established cardiac hypertrophy. (A) Schematic diagram of the CHAtRF anta injection and the experimental procedure. (B) qPCR results showing CHAtRF levels (*n* = 6 mice per group). (C) Top row: Analysis of cardiac morphology. Scale bar, 2 mm. Bottom row: Representative images of coronal sections of heart stained with hematoxylin and eosin. Scale bar, 2 mm. (D) HW/BW ratio (*n* = 8 to 9 mice per group). (E) Analysis of cardiomyocytes size (*n* = 6 to 8 mice per group). (F) qPCR results showing BNP mRNA levels (*n* = 6 mice per group). (G) Representative images of Masson,s trichrome-stained and semiquantitative analysis of histological sections of left ventricle. Scale bar, 20 μm (*n* = 6 to 8 mice per group). (H) Cardiac function measured by left ventricle ejection fraction (EF) using echocardiography (*n* = 6 to 8 mice per group). (I) Schematic diagram of CHAtRF function in hypertrophic signaling. CHAtRF participates in the regulation of cardiac hypertrophy by targeting the SRSF5/Psmg4 pathway. In our model, overexpression of CHAtRF prevents the binding of SRSF5 to Psmg4 pre-mRNA, resulting in AS of Psmg4 (exon 2 skipping) and a shift in Psmg4 full-length or short isoform expression, which subsequently promotes cardiac hypertrophy. Data are presented as mean ± SD. Data presented in (B) and (D) to (H) were analyzed by one-way ANOVA with Tukey post hoc test.

We next sought to test the therapeutic potential of CHAtRF inhibition in a model of established hypertrophy (Fig. 8A). CHAtRF inhibition (Fig. 8B) resulted in a reduction of hypertrophic responses as displayed by decreased heart weight/body weight ratios (Fig. 8C and D), cell size (Fig. 8E), expression of hypertrophic gene (Fig. 8F), and reduced cardiac fibrosis (Fig. 8G), as well as improved cardiac function (Fig. 8H) compared to animals treated with NC. The above results suggest that CHAtRF could be a therapeutic target for the treatment of pathological cardiac hypertrophy and potential HF.

We further investigated the hypertrophic effects of CHAtRF on human cardiomyocytes. Transfection of human induced pluripotent stem cell-derived cardiomyocytes (hiPSC-CMs) with a CHAtRF anta or NC (Fig. S9A) demonstrated that CHAtRF inhibition attenuated AngII-induced hypertrophy in these cells (Fig. S9B to D). Furthermore, CHAtRF overexpression led to decreased PSI of Psmg4 and increased exclusion of Psmg4 exon 2 (Fig. S9E), suggesting that CHAtRF promotes Psmg4 exon 2 skipping in hiPSC-CMs. To demonstrate the clinical relevance of CHAtRF, the serum samples from healthy individuals and individuals with hypertension and myocardial hypertrophy (Table S6) were collected to detect changes in CHAtRF levels. The content of CHAtRF in the serum of individuals with myocardial hypertrophy was significantly higher than that of healthy individuals (Fig. S9F to H). In addition, the expression levels of CHAtRF were significantly up-regulated in the serum of individuals with HF (Table S6) compared to those of healthy individuals (Fig. S9I to K). Taken together, these results indicate that CHAtRF might serve as a potential biomarker for diagnosing cardiac hypertrophy and HF.

## Discussion

Current research has identified over 500 distinct tRNAs in humans, with active gene expression accounting for nearly half of these molecules. tsRNAs exhibit high expression levels, evolutionary conservation, and structural stability while maintaining the functional diversity of tRNAs [33]. These molecules serve as epigenetic regulators, influencing mRNA stability, protein translation, and gene expression [34]. Emerging evidence confirms the involvement of tsRNAs in CVD progression, where they demonstrate significant regulatory capabilities. Recent studies have demonstrated that Tsr007330 modulates myocardial fibrosis development through its regulation of NAT10-dependent acetylation of EGR3 mRNA [35]. tsRNA-5008a induces cardiomyocyte ferroptosis, thereby contributing to atrial structural remodeling and increased susceptibility to atrial fibrillation [14]. However, whether tsRNAs can regulate pathological cardiac hypertrophy and their underlying molecular mechanisms remain poorly understood. In this study, we identified CHAtRF as a novel regulator of cardiac hypertrophy. Knockdown of CHAtRF alleviated AngII-induced cardiac hypertrophy and restored cardiac function, while forced expression of CHAtRF in cardiomyocytes caused a pathological hypertrophic response and led to impaired cardiac structure and function. Our study provides a new viewpoint for the role of tsRNAs in cardiac hypertrophy and reveals a novel pathogenic mechanism underlying cardiac hypertrophy and HF development. Our current study focuses on cardiomyocyte-autonomous mechanisms. Given that CHAtRF might be secreted via exosomes and tsRNAs are known to be involved in intercellular communication, investigating the potential paracrine effects of CHAtRF on noncardiomyocytes, such as cardiac fibroblasts, would provide valuable insights into its broader role in cardiac remodeling. This line of inquiry will be pursued in our future research.

The SRSFs represent a distinct class of RNA-binding proteins (RBPs) characterized by a C-terminal RS domain. As a member of the SR family, SRSF5 plays an important role in RNA splicing and protein translation, and its protein structure is conserved across different species [36,37]. Its C-terminal domain consists of arginine and serine amino acid sequences, primarily responsible for interactions between various proteins or RNA, while its N terminus contains 2 RNA recognition domains. Recent studies have found notable differential expression of SRSF5 in various cancers [38–40]. Experimental evidence has demonstrated that SRSF5 functions as a key regulator of AS for cyclin D binding myb like transcription factor 1 (DMTF1) pre-mRNA in colorectal cancer [39] and pyruvate kinase M1/2 (PKM) transcripts in breast cancer [41]. In addition, it is also crucial during heart development [24,42]. Nevertheless, the precise functional involvement and mechanistic basis of SRSF5 in pathological cardiac hypertrophy remain to be elucidated. Here, we generated cardiac-specific SRSF5 knockout mice and demonstrated that Srsf5-cKO mice enhanced AngII-induced hypertrophic effects. SRSF5 is able to directly bind with Psmg4 pre-mRNA and regulate the AS of Psmg4 during hypertrophy. To the best of our knowledge, this is the first study to elucidate the role and mechanism of SRSF5 in cardiac hypertrophy. However, whether SRSF5 can serve as a splicing factor for other precursor mRNAs (pre-mRNAs), such as DMTF1 or PKM in heart, remains to be further evaluated.

PSMG4 is a chaperone protein that plays a crucial role in promoting the correct assembly of the 20S proteasome and can affect protein degradation. PSMG4 and PSMG3 work synergistically to regulate protein turnover and maintain cellular homeostasis [25]. Research has found that PSMG family genes are closely related to immune response, ubiquinone metabolism, and cell cycle regulation [30]. Here, our results showed that overexpression of Psmg4 full-length (Psmg4-FL) isoform significantly inhibited AngII-induced hypertrophy in cardiomyocytes and in vivo relative to the short isoform, indicating that Psmg4 exon 2 inclusion is critical for the suppression of cardiac hypertrophy. Our results support the anti-hypertrophic functions of Psmg4-FL in cardiomyocytes, but its detailed regulatory mechanisms remain to be elucidated, representing a currently unaddressed knowledge gap in this study. Our preliminary investigation into downstream pathways revealed that overexpression of Psmg4-S attenuates proteasome activity, indicating that Psmg4-S overexpression may induce misfolded protein accumulation and cellular stress. We hypothesize that increased Psmg4-S, regulated by CHAtRF, disrupts proteasome assembly or function, leading to misfolded protein buildup and subsequent activation of stress pathways that promote cardiac hypertrophy. This avenue will be pursued in future research.

SRSF1 was the first member identified in 1990 as an SV40 pre-mRNA splicing regulator. Currently, 12 SRSF family members have been identified, typically containing 1 or 2 N-terminal RRMs coupled with the characteristic C-terminal RS domain [43,44]. The RIP-qPCR and biotin pull-down assays presented herein strongly support the direct binding of CHAtRF to SRSF5. To further confirm the specificity and binding affinity (*K*_d_) of this interaction and gain deeper mechanistic insight, we plan to employ electrophoretic mobility shift assays (EMSAs) or surface plasmon resonance (SPR). Currently, our laboratory faces technical and instrumental limitations preventing the immediate execution of these assays. However, we are committed to acquiring the necessary expertise and resources for their future implementation. In the current manuscript, we demonstrated that CHAtRF blocked SRSF5 to bind with Psmg4 pre-mRNA and promoted exon 2 skipping and AS of Psmg4 during hypertrophy. However, whether other members of the SRSF family besides SRSF5 are also involved in regulating Psmg4 AS remain to be further demonstrated. In addition, our results showed that the full-length isoform of Psmg4 attenuated AngII-induced hypertrophic effects relative to short isoform. Different pressure stimuli can induce cardiac hypertrophy, and further identification is needed to determine whether the short isoform of Psmg4 has inhibitory effects on hypertrophy under other pressure stimuli.

Our study demonstrates that CHAtRF critically regulates cardiac hypertrophy by controlling Psmg4 AS. These findings uncover a novel tsRNA-mediated mechanism in hypertrophic pathogenesis and advance our understanding of splicing regulation in cardiac remodeling, revealing potential therapeutic targets for pathological cardiac hypertrophy and HF treatment.

## Materials and Methods

### Cell culture and transfection

The neonatal mouse ventricular cardiomyocytes were isolated from newborn mice (1 to 2 days old). Mouse hearts were washed with phosphate-buffered saline (PBS), cut into small pieces, and placed in a digest containing collagenase (0.14 mg/ml) and trypsin (1.2 mg/ml). Digestion was performed at 37 °C, with shaking for 5 min each time, and fetal bovine serum was used to terminate digestion. Digestion was repeated 6 to 10 times, and cells were collected by centrifugation. Cardiomyocytes and fibroblasts were isolated by differential adhesion in serum-free Dulbecco,s modified Eagle,s medium (DMEM)/F12 medium (MeilunBio, MA0214) for 1 to 2 h. Cardiomyocytes were cultured in DMEM/F12 medium containing 1% 5-bromo-2′-deoxyuridine and 5% serum for 24 h and used for subsequent experiments. We confirm that mouse cardiomyocytes and fibroblasts were contamination free.

Cardiomyocyte hypertrophy was induced by treatment with 1 μM AngII for 48 h. For cell transfection, a mixture of Srsf5 small interfering RNAs (siRNAs) (Genepharma, Shanghai, China) or NC was transfected with Lipofectamine 8000 Reagent (Beyotime Biotechnology, C0533) under the manufacturer,s instructions. Srsf5-siRNA (si-Srsf5): sense, 5′-GGAUGCACAUCGACCUAAATT-3′; anti-sense, 5′-UUUAGGUCGAUGUGCAUCCTT-3′. When agomir or anta (Genepharma, Shanghai, China) was transfected, the transfection method was used as the siRNA transfection method. Cardiomyocytes from human induced pluripotent stem cells (hiPSC-CMs) were obtained from NanJing Cosmos Biotechnology Co. Ltd.

### Animal studies

Adult C57BL/6 mice (8 to 10 weeks old) were sourced from Jinan Pengyue Laboratory Animal Breeding Co. Ltd. (Jinan, Shandong). The study adhered to an experimental protocol approved by the Animal Protection Committee of Qingdao University and complied with the National Institutes of Health (NIH) Guide for the Care and Use of Laboratory Animals. Randomization was applied throughout the study, with investigators blinded via numerical coding of mice and samples. No animals were excluded, and analyses were restricted to mice with comparable body weights and normal physiological indices during surgical procedures.

### Generation of Srsf5-cKO mice

The *Srsf5*-cKO mouse model was created by Cyagen Biosciences Inc. (China) employing CRISPR/Cas9 genome editing. Srsf5^flox/flox^ mice were crossbred with Myh6^Cre^ mice (purchased from Saiye Model Organisms) to generate Myh6^Cre^-Srsf5^flox/flox^ (termed SRSF5-cKO) mice. Srsf5^flox/flox^ littermates served as controls (Ctr). All mice were maintained in the C57BL/6 background, and 8- to 10-week-old male mice were used in this study. Neonatal mice will be genotyped by PCR followed by sequencing analysis.

For Psmg4 knockdown or overexpression in vivo, the vector was constructed by adenovirus (Genepharma, Shanghai, China). The adult mice were injected with adenovirus (2 × 10^10^ moi) harboring Psmg4-S, Psmg4-FL, or shPsmg4 vectors by tail vein injection 5 to 7 days before saline or AngII (Abmole, A107852) treatment. After AngII (2 mg/kg) treatment for 2 weeks, heart samples were collected to conduct molecular and morphological studies.

### The synthesis of CHAtRF anta and injection in vivo

CHAtRF anta was synthesized by Genepharma (Shanghai). The synthesized anta was 5′-UGGCAACCACGAAGGGAU-3′. The synthesized NC (anta-NC) was 5′-CAGUACUUUUGUGUAGUACAA-3′. The 3′ end was modified with cholesterol and 4 thioskeletons. Two thioskeletons at the 5′ end are modified to improve the ability of chain stability to enter cells. Modification of 2′-O-Me was performed in the whole chain to increase affinity and avoid degradation. Anta or anta-NC (30 mg/kg) was injected twice via tail vein to knock down CHAtRF in vivo*.*

### RNA isolation and RT-qPCR

Total RNA was extracted from cells or tissues using Trizol reagent (Vazyme) under low-temperature conditions. Briefly, 1 ml of Trizol was added to the sample and incubated for 5 min at room temperature. Then, 200 μl of chloroform was added, followed by vigorous shaking for 15 s. The mixture was centrifuged at 12,000 rpm for 15 min at 4°C, and the upper aqueous phase was carefully collected. An equal volume of isopropanol was added, and the sample was incubated at −20°C for 30 min to facilitate RNA precipitation. After centrifugation at 12,000 rpm for 15 min at 4°C, the RNA pellet was collected and washed with 1 ml of precooled 75% ethanol. Following a final centrifugation at 12,000 rpm for 10 min at 4°C, the supernatant was discarded, and the pellet was air-dried for 5 to 10 min. Finally, the RNA was dissolved in RNase-free water, and its concentration and purity were determined using a NanoDrop spectrophotometer.

RNA was reverse transcribed using the Evo M-MLV RT Kit (Accurate Biology, AG11705) to yield cDNA. Then, the qPCR kit (Yeasen, 11202ES60) was used for RT-qPCR amplification, the cycle threshold (Ct) value was read on a real-time PCR instrument, and the relative expression level was calculated using the ∆∆Ct method. The specific primers used to RT-qPCR are provided in Table S5.

### Protein extraction and WB

Total protein was extracted from cells or tissues using radioimmunoprecipitation assay (RIPA) (Solarbio, R0010) at low-temperature conditions. Briefly, the samples were lysed with RIPA (containing 1% phenylmethylsulfonyl fluoride) for 30 to 45 min. Then, the samples were centrifuged at 12,000 rpm for 15 min, and the supernatant was collected and mixed with ^1^/_4_ volume of 5× loading buffer (Solarbio, P1040) before heating at 99 °C for 10 min. The collected protein sample was loaded onto sodium dodecyl sulfate–polyacrylamide gel electrophoresis (SDS-PAGE) gel for electrophoresis at 80 V for 30 min and 120 V for 60 min, and then transferred to a polyvinylidene difluoride (PVDF) membrane via electrotransfer for 80 to 120 min. After blocking with 5% skim milk for 1 to 2 h at room temperature, the antibody was used for incubation. After cleaning the PVDF membranes with phosphate-buffered saline with Tween 20 (PBST), the PVDF membranes were briefly incubated with enhanced chemiluminescence (ECL) luminescent solution (Vazyme, E422-02) to collect signals. Antibodies used for WB included anti-cardiac Troponin T (anti-cTnT) (Bioss, bs-10648), anti-SRSF5 (Proteintech, 16237-1-AP), anti-glyceraldehyde-3-phosphate dehydrogenase (GAPDH) (ABclonal, A19056), and anti-PSMG4 (Invitrogen, PA5-66942).

### Fluorescence in situ hybridization

The subcellular localization of CHAtRF in cardiomyocytes was detected according to the instructions of the fluorescence in situ hybridization (FISH) kit (Genepharma, Shanghai, China). Briefly, cells or tissue slices were immersed in 4% paraformaldehyde and fixed at room temperature for 15 min. After washing with PBS buffer, 0.1% Buffer A was added and incubated at room temperature for 15 min. After washing with PBS buffer, 2× Buffer C was added and incubated at 37 °C for 30 min. Then, 70%, 80%, 95%, and 100% ethanol were added for gradient dehydration. Cells or tissue slices were incubated overnight at 37 °C with fluorescent probes that specifically bound to intracellular CHAtRF. Then, 0.1% Buffer F was added to wash, followed by 2× Buffer C. Nuclei were labeled using 4′,6-diamidino-2-phenylindole (DAPI), and signals were collected by confocal laser scanning microscopy.

### Proteasome activity assay

Proteasome activity was measured using the fluorogenic substrate LLVY-AMC according to the manufacturer,s instructions. Briefly, LLVY-AMC substrate and MG-132 inhibitor stock solutions were prepared as recommended. Cell or tissue lysates were generated using the provided Assay Buffer, clarified by centrifugation, and the protein concentration was determined. Reactions were set up in a black 96-well plate by adding lysates containing 10 to 50 μg of total protein to each well and adjusting the volume to 100 μl with Assay Buffer. For specificity controls, lysate aliquots were preincubated with MG-132 (final concentration 10 to 20 μM). The reaction was initiated by adding 2 μl of LLVY-AMC substrate stock solution [10 mM in dimethyl sulfoxide (DMSO)] to each well, except for blank wells containing DMSO only, yielding a final substrate concentration of approximately 200 μM. Fluorescence was measured kinetically at 37 °C using a microplate reader (excitation/emission = 350/440 nm) at 5-min intervals for 30 to 60 min. Proteasome activity was calculated as the relative fluorescence units (RFU/min) after subtraction of blank and inhibitor-treated control values.

### Hematoxylin and eosin staining

Tissue sections were stained according to the instructions of the hematoxylin and eosin (H&E) kit (Solarbio, G1120). In brief, paraffin sections were dewaxed and rehydrated after passing through a series of gradient ethanol solutions. The tissue sections were stained with hematoxylin for 5 to 10 min, followed by differentiation in a differentiation solution for 2 min. After differentiation, the sections were rinsed twice with running tap water for 1 min each. Subsequently, they were counterstained with eosin for 1 to 2 min. Dehydration was then performed using a graded ethanol series, followed by clearing in xylene, and finally mounted in glycerol–gelatin. The images were observed and photographed under an optical microscope.

### WGA staining

Frozen tissue sections were placed at room temperature for 10 min. They were then washed with PBS and fixed with 4% paraformaldehyde (Biosharp) for 25 min. After washing with PBS again, the sections were stained with IFluor 488-WGA (Solarbio, I3300) in the dark for 30 min. After washing with PBS, the slides were stained with DAPI (Solarbio, C0065) and sealed. Paraffin sections were dewaxed and rehydrated after passing through a series of gradient ethanol solutions. The staining steps are the same as those for frozen sections. The images were observed and photographed under a confocal laser scanning microscope. Cardiomyocyte cross-sectional area was quantified using ImageJ.

### Masson staining

Masson staining was performed according to the instructions of the Masson,s Trichrome Stain Kit (Solarbio, G1340). In brief, paraffin sections were dewaxed and rehydrated after passing through a series of gradient ethanol solutions. The sections were stained with Weigert,s iron hematoxylin solution for 8 min. The sections were treated with acid alcohol differentiation solution for 10 s and washed with water for 1 min. Then, the sections were treated with blue in bluing solution for 4 min and washed with water for 1 min. The sections were stained with ponceau-acid fucshin solution for 8 min and washed with acetic acid working solution for 1 min. The sections were treated with phosphomolybdic acid solution for 1 to 2 min and then cleaned with acetic acid working solution for 1 min. The sections were stained with aniline blue solution for 10 to 30 s and washed with acetic acid working solution for 1 min. After rapid dehydration through a gradient alcohol solution, the slices were transparent with xylene, sealed with glycerin gelatin, and photographed for observation under a microscope. The myocardial tissue appears red, while collagen appears blue. Quantitative statistics of fibrosis area were performed using ImageJ.

### TAC model

Typically performed on 8- to 10-week-old mice, the procedure begins with anesthesia induction using isoflurane, followed by endotracheal intubation and mechanical ventilation. After a midline cervical incision and careful dissection of the overlying muscles, the transverse aortic arch is isolated between the brachiocephalic and left common carotid arteries. A ligature (7-0 or 8-0 suture) is passed underneath the aorta and tied tightly against a 27-gauge needle or a blunted needle segment of defined diameter placed alongside the vessel. The needle is then promptly removed, creating a standardized, critical stenosis. Following ligation, the muscle and skin layers are sutured closed. Post-operative analgesia, such as buprenorphine, is administered, and mice are monitored closely during recovery in a warm environment. Successful pressure overload is validated 2 weeks post-surgery via transthoracic echocardiography, which reveals characteristic increases in left ventricular wall thickness and mass, along with eventual systolic dysfunction compared to sham-operated controls.

### Mouse swimming training

The procedure begins by preparing buckets with warm water, ensuring that the temperature, monitored by a submerged thermometer probe, is maintained at 30 to 32 °C with a depth of approximately 15 cm, using a net for adjustment. Mice are gently placed into the water at a density of 12 per bucket, followed by immediate timer initiation. Throughout the session, mice are closely monitored: Improper swimming postures are corrected with a net, fecal matter is promptly removed, and water temperature is consistently regulated by adding hot water as needed. Mice showing exhaustion or choking are immediately rescued, allowed a brief rest on the net, and then returned to the water under continuous supervision to prevent drowning. Upon timer completion, mice are netted, dried with paper towels, transferred to a drier cage, and thoroughly dried using a hair dryer on the lowest setting for approximately 20 min. After drying, they are housed in clean cages with ample fresh food and water before being returned to the rack. Training is conducted twice daily (8:30 to 10:00 AM and 3:00 to 4:30 PM) with a 4- to 6-h interval. The regimen starts on day 0 with 5-min swims, incrementally increasing daily (e.g., 10 min on day 1, 20 min on day 2) until reaching a fixed duration of 90 min per session by day 9, which is then sustained for all subsequent sessions to ensure consistent endurance training with adequate recovery between daily bouts.

### Echocardiographic assessment

After the completion of the experiment, conscious mice were restrained or fixed on a heating plate at 37 °C for a short time. The hair in the detection area was removed with a cotton swab dipped in depilation cream, and the cardiac function was evaluated by echocardiography using a small-animal ultrasound imaging system (VINNO 6 LAB). Isoflurane at a concentration of 2% was used to anesthetize the mice, and anesthesia was maintained with 1% to 1.5% isoflurane during image acquisition. Left ventricular EF, FS, and heart rate were monitored by M-mode echocardiography. The E/A ratio was monitored by pulsed wave Doppler mode (PW-mode) of echocardiography. All the experiments were blinded, and at least 3 beats were averaged for all measurements.

### Phalloidin staining

Phalloidin can label actin F-actin in cardiomyocytes, enabling quantitative analysis of cell area. The cultured cardiomyocytes were washed with PBS and fixed in 4% paraformaldehyde for 25 min at room temperature. After washing with PBS, the cells were treated with 0.1% to 0.5% Triton X-100 (MeilunBio, MB2486) for 5 min. Subsequently, the cells were washed with PBS and incubated with phalloidin probe (Yeasen, 40734ES80) for 30 min at room temperature in the dark. After washing with PBS, DAPI staining was performed, and the slides were sealed. The images were observed and photographed under a confocal microscope. Cardiomyocyte surface area was analyzed using ImageJ.

### RNA pulldown and LC-MS/MS analysis

Cardiomyocytes were digested with lysis buffer (containing 20 mM tris–HCl, 200 mM NaCl, 2.5 mM MgCl_2_, 1 mM dithiothreitol, and 1% protease inhibitor) for 30 min. Lysates were centrifuged, and the remaining 10% lysate was used as input. The remaining 90% lysates were incubated with biotin probes and beads for 12 to 16 h. After washing the beads clean, they were boiled in 2× loading buffer. After SDS-PAGE gel electrophoresis and Coomassie blue staining, the pull-down samples of CHAtRF and NC were collected and analyzed by liquid chromatography–tandem mass spectrometry (LC-MS/MS) by Applied Protein Technology (Shanghai).

### tsRNA microarray analysis

The tsRNA microarray analysis was performed by Aksomics Biotech Ltd. (Shanghai, China). Total RNA was extracted from cardiac tissues of both TAC-operated hypertrophic mice and sham control mice. The concentration of RNA was measured with a NanoDrop ND-1000 spectrophotometer, while its integrity was assessed using either a Bioanalyzer 2100 or agarose gel electrophoresis under denaturing conditions. For the tsRNA microarray analysis by Arraystar, 100 ng of RNA was dephosphorylated at 37 °C for 40 min with 3× T4 polynucleotide kinase. This process involved the removal of phosphoric (P) and cyclic phosphoric (cP) chemical groups at the 3′ end of the RNA, resulting in the formation of the 3-OH end. The 5′ ends of the RNA were labeled with pCp-Cy3 at 16 °C overnight in a solution containing ligase buffer, bovine serum albumin, and T4 RNA ligase. The labeled RNA was then hybridized with the hybridization buffer at 100 °C for 5 min before being chilled to 0 °C. The hybridization of the 45-μl labeled RNA sample to the microarray was carried out at 55 °C for 20 h. Sample mixtures were scanned using an Agilent G2505C microarray scanner, and the images were processed with Agilent Feature Extraction software (version 11.0.1.1). The data underwent log_2_ transformation and normalization. Only samples with probe signals marked as present (P) or marginal (M) by the quality control metrics were considered for further analyses. The differential expression of tsRNAs was determined by comparing the fold change (FC) and statistical significance (*P* value) between the 2 groups.

### RNA immunoprecipitation and RIP-seq

The NC- or anta-transfected cells were lysed and then immunoprecipitated with anti-SRSF5 antibody (Proteintech, 16237-1-AP) or anti-immunoglobulin G (IgG) (Abclonal, China) using rProtein A/G agarose resin (Yeasen, 36403ES08). After an overnight incubation at 4 °C, the protein A/G agarose beads were added, and then the solution was incubated for 4 h at 4 °C. The beads were washed 3 times with PBS and resuspended in lysis buffer. The remaining products were extracted with Trizol, and the levels of RNA were quantified by RT-qPCR. RIP-seq assays were conducted by Kang Chen Bio-tech (Shanghai, China). Genes with an adjusted *P* value of <0.05 [after false discovery rate (FDR) correction] and an absolute FC of >2 were classified as differentially expressed.

### Human heart samples

The study utilized human myocardium samples collected at Tongji Hospital (Wuhan, China), with ethical approval granted by the institutional review boards of both Tongji Hospital and Tongji Medical College. Normal myocardium samples were derived from autopsy cases of traffic accident victims lacking CVD history, processed through the Department of Forensic Medicine at Tongji Medical College. All procedures complied with the Helsinki Declaration, with written informed consent provided by the relatives of deceased. Myocardial specimens were obtained from HCM patients during modified Morrow septal myectomy procedures. Before sample collection, written informed consent was secured from either the patients themselves or their closest relatives. This research, conducted in compliance with the Declaration of Helsinki guidelines, was ethically approved by Beijing Anzhen Hospital,s institutional review board (affiliated with Capital Medical University).

### Human plasma samples

A total of 15 patients diagnosed with cardiac hypertrophy and 15 patients with heart failure were recruited for this study, conducted at Anzhen Hospital, Capital Medical University, Beijing, between October 2024 and January 2025. The study was prospective in design and received approval from the Institutional Review Board. All procedures adhered to the ethical principles outlined in the Declaration of Helsinki. Written informed consent was obtained from each participant prior to enrollment. Following an overnight fast, blood samples were collected in tubes containing ethylenediamine tetraacetic acid (EDTA) and plasma samples were stored at −80 °C.

### Statistical analysis

All data are presented as mean ± SD. GraphPad Prism (GraphPad Software Inc., San Diego, CA) was used for statistical analysis. We confirm that both normality tests and homogeneity of variance tests were conducted prior to performing our statistical analyses. For data with normal distribution, unpaired Student,s *t* test (2-tailed) was used for 2 groups, and one-way analysis of variance (ANOVA) with Tukey multiple comparisons test was used for multiple groups (one variable). Normality distributed data with 2 variables were evaluated by 2-way ANOVA with Tukey multiple comparisons test. Experiments were repeated independently at least 3 times, with similar results. A *P* value of <0.05 was considered statistically significant.

## Data Availability

Data supporting the findings of this study are available from the corresponding authors upon reasonable request.
