# Supplementary material for: CHAtRF Modulates Cardiac Hypertrophy via SRSF5-Dependent Regulation of Psmg4 Alternative Splicing
Source: Research (Wash D C). 2026 Mar 26;9:1202. doi: 10.34133/research.1202 (PMC13018653; doi:10.34133/research.1202)
Supplement: Supplementary 1 — Figs. S1 to S9 Tables S1 to S7 [file research.1202.f1.zip › Table S6.docx]

| Clinical information | | Normal controls  （N=15） | | Patients with hypertrophy（N=15） | | Patients with heart failure（N=15） | |
| --- | --- | --- | --- | --- | --- | --- | --- |
| Age(years) | | 46.8±7.2 | | 48.1±9.5 | | 49.9±12.1 | |
| Male (%) | | 73.3 | | 73.3 | | 73.3 | |
| Etiology | | / | |  | |  | |
| Hypertension (%) | | / | | 100 | | 66.7 | |
| Coronary heart disease (%) | | / | | 0 | | 20 | |
| Dilated cardiomyopathy (%) | | / | | 0 | | 13.3 | |
| NYHA class | | / | | / | |  | |
| Class II (%) | |  | |  | | 60 | |
| Class III (%) | |  | |  | | 40 | |
| Medication | | / | |  | |  | |
| Loop diuretics (%) | |  | | 0 | | 33.3 | |
| ACEI/ARB/ARNI (%) | |  | | 46.7 | | 66.7 | |
| ß-Blockers (%) | |  | | 6.7 | | 66.7 | |
| MRA (%) | |  | | 0 | | 20 | |
| CCB (%) | |  | | 0.8 | | 66.7 | |

ACEI，Angiotensin-Converting Enzyme Inhibitor；ARB，Angiotensin II Receptor Blocker；ARNI，Angiotensin Receptor-Neprilysin Inhibitor；MRA：Mineralocorticoid Receptor Antagonist；CCB，Calcium Channel Blocker

Age did not differ significantly between the normal controls and patients with hypertrophy （p=o.668）, nor between the normal controls and those with heart failure （p=o.396）. The distribution of gender was balanced among the three groups.
