# Supplementary material for: CHAtRF Modulates Cardiac Hypertrophy via SRSF5-Dependent Regulation of Psmg4 Alternative Splicing
Source: Research (Wash D C). 2026 Mar 26;9:1202. doi: 10.34133/research.1202 (PMC13018653; doi:10.34133/research.1202)

Supplementary Materials

Supplementary Figure legends

Figure S1. Identification of tsRNAs in cardiomyocytes with TAC or AngII stimulation. (A) The expression levels of tsRNAs in TAC-treated mice hearts analyzed by qPCR (n=6 mice per group). (B) The expression levels of tsRNAs in AngII-treated cardiomyocytes analyzed by qPCR (n=6 independent experiments). (C) The schematic of the origin of tRF3a-ArgTCG-3. Data are presented as Mean±SD. Data presented in A and B were analyzed via 2-way ANOVA with Tukey post hoc test.

Figure S2. CHAtRF deficiency attenuates pathological cardiac hypertrophy caused by AngII. (A) Mice were injected with CHAtRF antagomir (anta) or its negative control (NC). The expression levels of CHAtRF were analyzed by qPCR (n=6 mice per group). (B-G) Mice were injected with CHAtRF antagomir (anta) or its negative control (NC) and then they were treated with AngII. (B) Heart weight (HW) to tibia length (TL) ratio (n=6 mice per group). (C) QPCR analysis of the expression level of ANP mRNA (n=6 mice per group). (D-G) Heart rate, ejection fraction, fractional shortening and E/A ratio detected by echocardiography in mice (n=6 mice per group). Data are presented as Mean±SD. Data presented in A, B, C, D, E, F and G were analyzed via one-way ANOVA with Tukey post hoc test.

Figure S3. CHAtRF deficiency alleviate the cardiac hypertrophy caused by TAC. (A-E) Mice were injected with CHAtRF antagomir (anta) or its negative control (NC) and then they were subjected to TAC modeling. (A) Representative images of gross morphology of hearts (upper row). Bar=2mm. Representative images of left ventricular muscle sections stained with WGA (bottom row). Bar=25μm. (B) Heart weight (HW) to body weight (BW) ratio (n=6 mice per group). (C) Analysis of the cardiomyocyte sizes in histological sections stained with WGA (n=6 mice per group). (D-E) qPCR

analysis of the expression levels of BNP mRNA and β-MHC mRNA (n=6 mice per group). (F) Cardiomyocytes were transfected with CHAtRF agomir (CHAtRF) or its negative control (NC) for 24 h. Changes in CHAtRF expression at different time points after Ang II treatment (n=6 independent experiments). Data are presented as Mean±SD. Data presented in B, C, D and E was analyzed via 2-way ANOVA with Tukey post hoc test. Data presented in F was analyzed via one-way ANOVA with Tukey post hoc test.

Figure S4. CHAtRF binds to SRSF5. (A) The CHAtRF and NC pull-down samples were loaded into the SDS-PAGE gel and were dyed with coomassie brilliant blue dye after electrophoresis. (B) QPCR (upper panel) and western blotting (lower panel) analyses of SRSF5 levels in cardiomyocytes transfected with CHAtRF and NC (n=4 independent experiments). (C) QPCR (upper panel) and western blotting (lower panel) analyses of SRSF5 levels in cardiomyocytes transfected with anta and anta-NC (n=4 independent experiments). (D) Scatter plot of differential expression of RNAs assessed from RNA-seq data in cardiomyocytes transfected with CHAtRF antagomir (anta) or NC. Red dots denote up-regulated genes and green dots denote down-regulated genes. (E) After TAC modeling, RT-PCR analysis for AS event of Psmg4 gene in NC and antagomir cardiomyocytes. (n=6 independent experiments). (F) RT-PCR analysis for AS event of Psmg4 gene in GFP and SRSF5-ovexpressed cardiomyocytes. (n=6 independent experiments). Data are presented as Mean±SD. Data presented in B, C and F were analyzed via one-way ANOVA with Tukey post hoc test. Data presented in E was analyzed via 2-way ANOVA with Tukey post hoc test.

Figure S5. Psmg4 full length isoform inhibits AngII-induced hypertrophy. (A-E) Adenovirus harboring Psmg4-S, Psmg4-FL and CTRL were injected into 8-10 week old mice, and AngII was treated after 1 week to induce cardiac hypertrophy. Heart samples were collected 2 weeks post-AngII treatment. (A) The expression levels of Psmg4 mRNA in hearts (n=6 mice per group). (B) Heart weight (HW) to tibia length (TL) ratio (n=6 mice per group). (C-E) Heart rate, fractional shortening and E/A ratio detected by echocardiography in mice (n=6 mice per group). (F) Cardiomyocytes were

infected with adenovirus harboring Psmg4-S, Psmg4-FL and CTRL for 24 h. The activity of the proteasome under different treatments (n=6 independent experiments). Data are presented as Mean±SD. Data presented in A-F were analyzed via one-way ANOVA with Tukey post hoc test.

Figure S6. Knocking down Psmg4 inhibits the therapeutic effect of CHAtRF antagomir against cardiac hypertrophy. (A-C) Mice were treated with Ang II and transfected with CHAtRF antagomir or its NC, while infected with adenovirus harboring shPsmg4 or shCTRL. (A) QPCR analysis of the expression level of BNP mRNA (n=6 mice per group). (B) Analysis of LV collagen volume fraction (n=6 mice per group). (C) Left ventricular EF detected by echocardiography in mice (n=6 mice per group). (D) Mice were injected with adenovirus harboring shPsmg4 or shCTRL. QPCR analysis of the expression level of Psmg4 mRNA (n=6 mice per group). (E) Mice were treated as described in (A). QPCR analysis of the expression level of Psmg4 mRNA (n=6 mice per group). Data are presented as Mean±SD. Data presented in A-E were analyzed via one-way ANOVA with Tukey post hoc test.

Figure S7. Identification of Srsf5-cKO mice. (A) The expression level of SRSF5 mRNA in SRSF5$^{fl/fl}$ mice and SRSF5-cKO mice hearts analyzed by qPCR (n=12 mice per group). (B) The expression level of BNP mRNA in SRSF5$^{fl/fl}$ mice and SRSF5 cKO mice hearts (n=9 mice per group). (C) Left ventricular EF detected by echocardiography in SRSF5$^{fl/fl}$ mice and SRSF5-cKO mice (n=9 mice per group). (D-I) Srsf5-cKO mice were injected with AngII. (D) Representative images of gross morphology of hearts (upper row). Bar=2mm. Representative images of left ventricular muscle sections stained with WGA (middle row). Bar=25μm. Representative images of Masson's trichrome-stained histological sections (bottom row). Bar=20μm. (E) Heart weight (HW) to body weight (BW) ratio (n=7-8 mice per group). (F) Analysis of the cardiomyocyte sizes in histological sections stained with WGA (n=7 mice per group). (G) Quantitative analysis of collagen contents in left ventricle samples (n=6 mice per group). (H) QPCR analysis of the expression level of BNP mRNA (n=6 mice per group).

(I) Left ventricular EF detected by echocardiography in mice (n=6 mice per group). (J) Srsf5-cKO mice were used to establish the TAC model. Heart weight (HW) to body weight (BW) ratio (n=7 mice per group). Data are presented as Mean±SD. Data presented in A, B and C were analyzed via Student t test (2-tailed). Data presented in E-J were analyzed via 2-way ANOVA with Tukey post hoc test.

Figure S8. CHAtRF deficiency fails to ameliorate swim training-induced cardiac hypertrophy. (A-H) Mice were injected with CHAtRF antagomir (anta) or its negative control (NC) and then they underwent swimming training. (A) Schematic diagram of the animal model. (B) Representative images of gross morphology of hearts (upper row). Bar=2mm. Representative images of left ventricular muscle sections stained with WGA (bottom row). Bar=25μm. (C) Heart weight (HW) to body weight (BW) ratio (n=6 mice per group). (D) Heart weight (HW) to tibia length (TL) ratio (n=6 mice per group). (E) Analysis of the cardiomyocyte sizes in histological sections stained with WGA (n=6 mice per group). (F) Analysis of Ki67-positive cells in tissue sections with swim model or control (n=6 mice per group). (G) Quantification of SRSF5 protein expression by Western blot (n=6 mice per group). (H) The quantification of splicing percentages. Data are presented as Mean±SD. Data presented in G was analyzed via Student t test (2-tailed). Data presented in C-F and H were analyzed via 2-way ANOVA with Tukey post hoc test.

Figure S9. The effects of CHAtRF on human induced pluripotent stem cell-derived cardiomyocytes (hiPSC-CMs). (A) HiPSC-CMs were transfected with CHAtRF antagomir (anta) or its NC. CHAtRF levels were analyzed by qRT-PCR (n=6 independent experiments). (B-D) HiPSC-CMs were transfected with CHAtRF antagomir (anta) or its NC, and then were treated with AngII. (B) Quantitative analysis of the cell surface area was assessed (n=7-8 independent experiments). (C) QPCR analysis of the expression level of ANP mRNA (n=6 independent experiments). (D) QPCR analysis of the expression level of BNP mRNA (n=6 independent experiments). (E) RT-PCR analysis for AS event of Psmg4 gene in NC and CHAtRF-overexpressing

hiPSC-CMs. (n=6 independent experiments). (F-K) The expression of human CHAtRF in sera of individuals with cardiac hypertrophy (F, n=15) or HF (I, n=15). HF, heart failure. Subsequently, the tests were conducted separately based on gender. (G and J, Female, n=4; H and K, Male, n=11). Data are presented as Mean±SD. Data presented in A-K were analyzed via Student t test (2-tailed).

Supplementary Figure 1

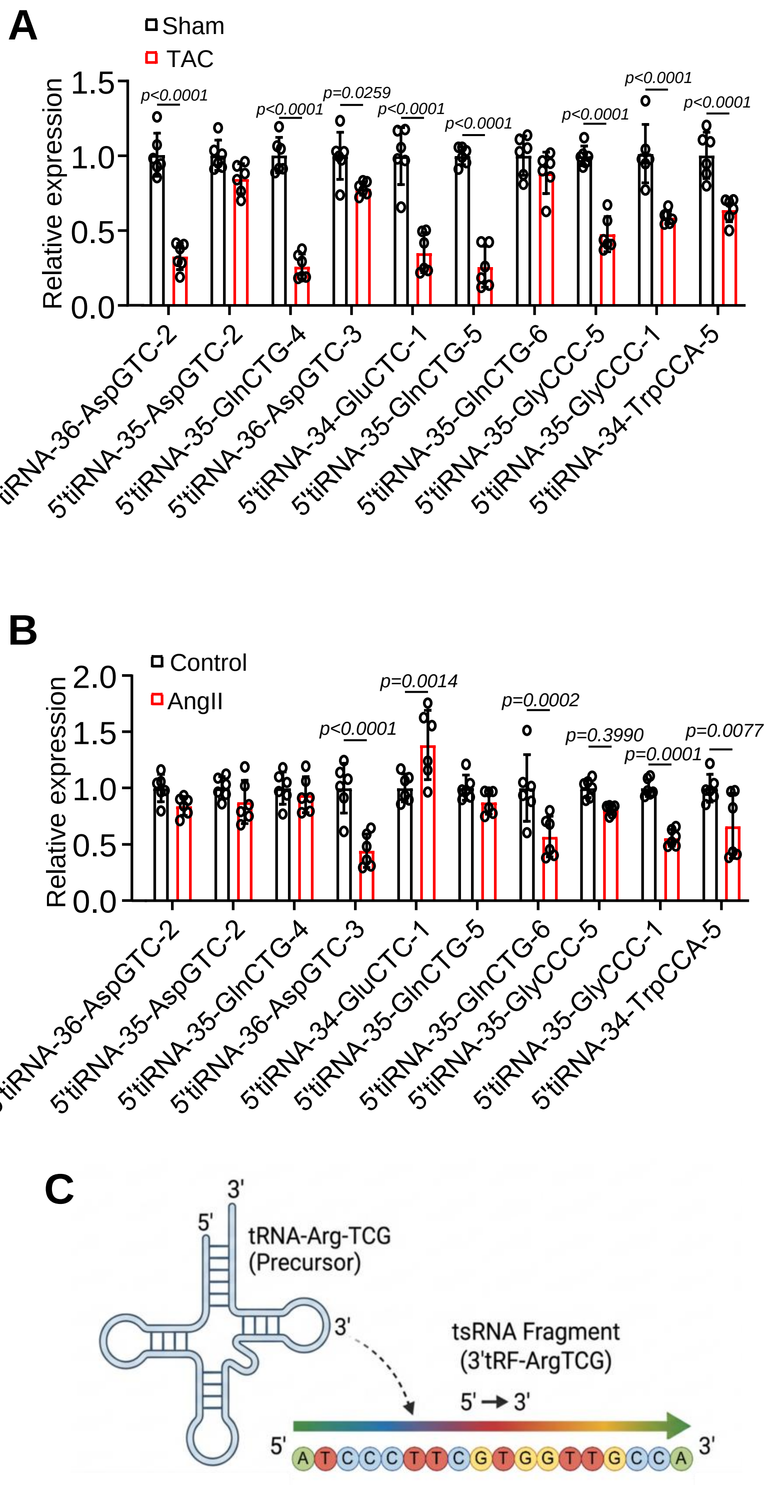

Supplementary Figure 2

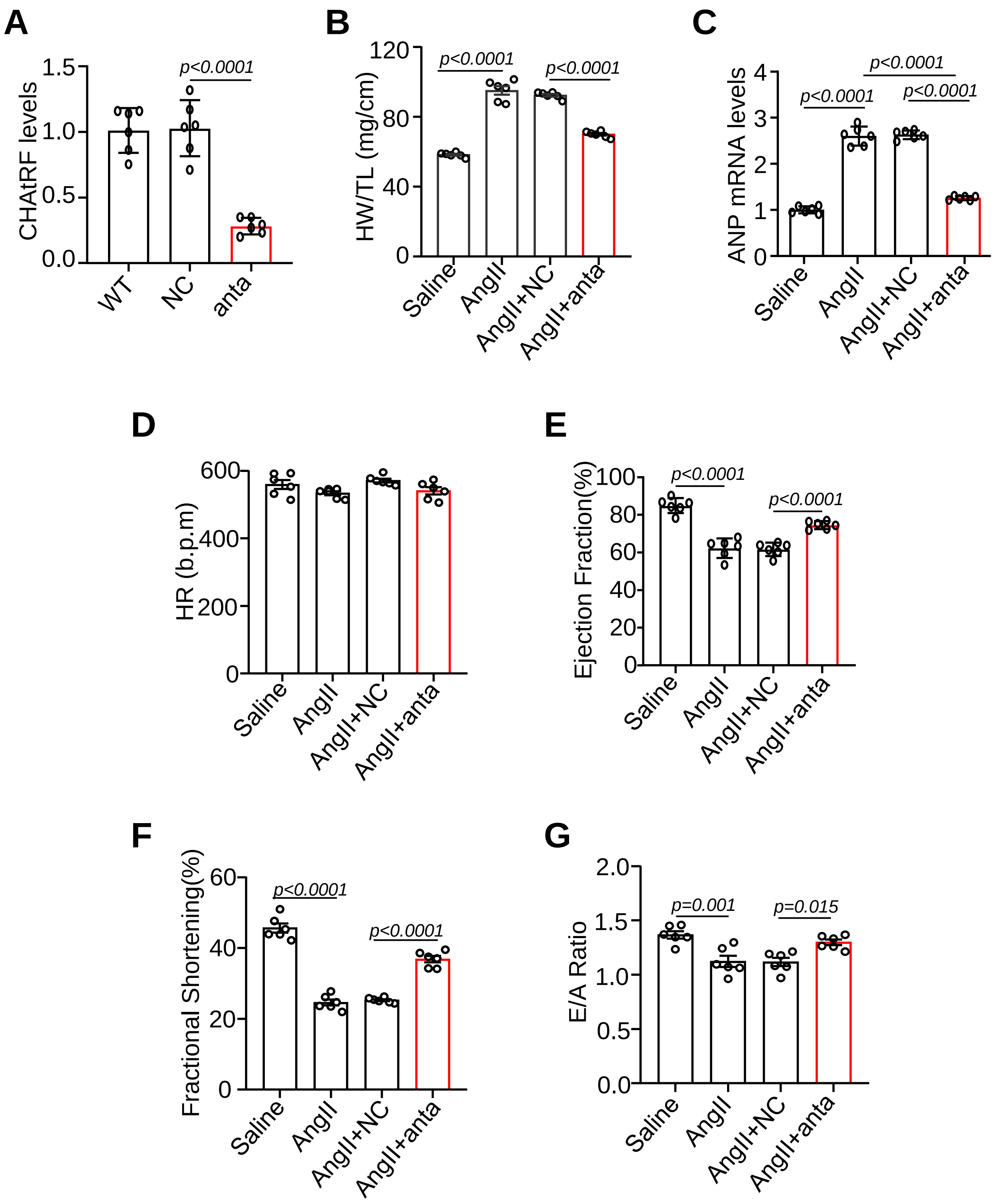

Supplementary Figure 3

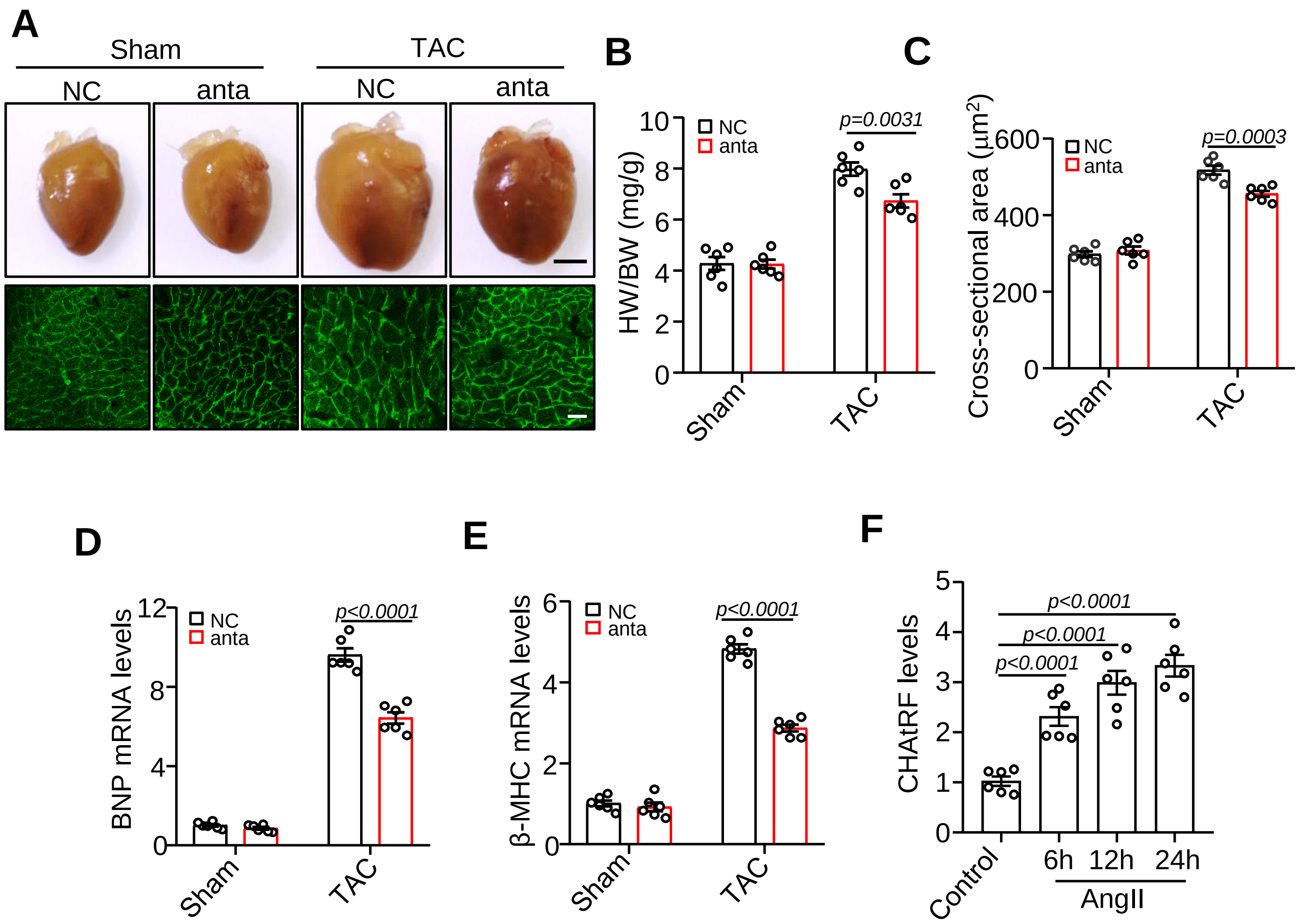

Supplementary Figure 4

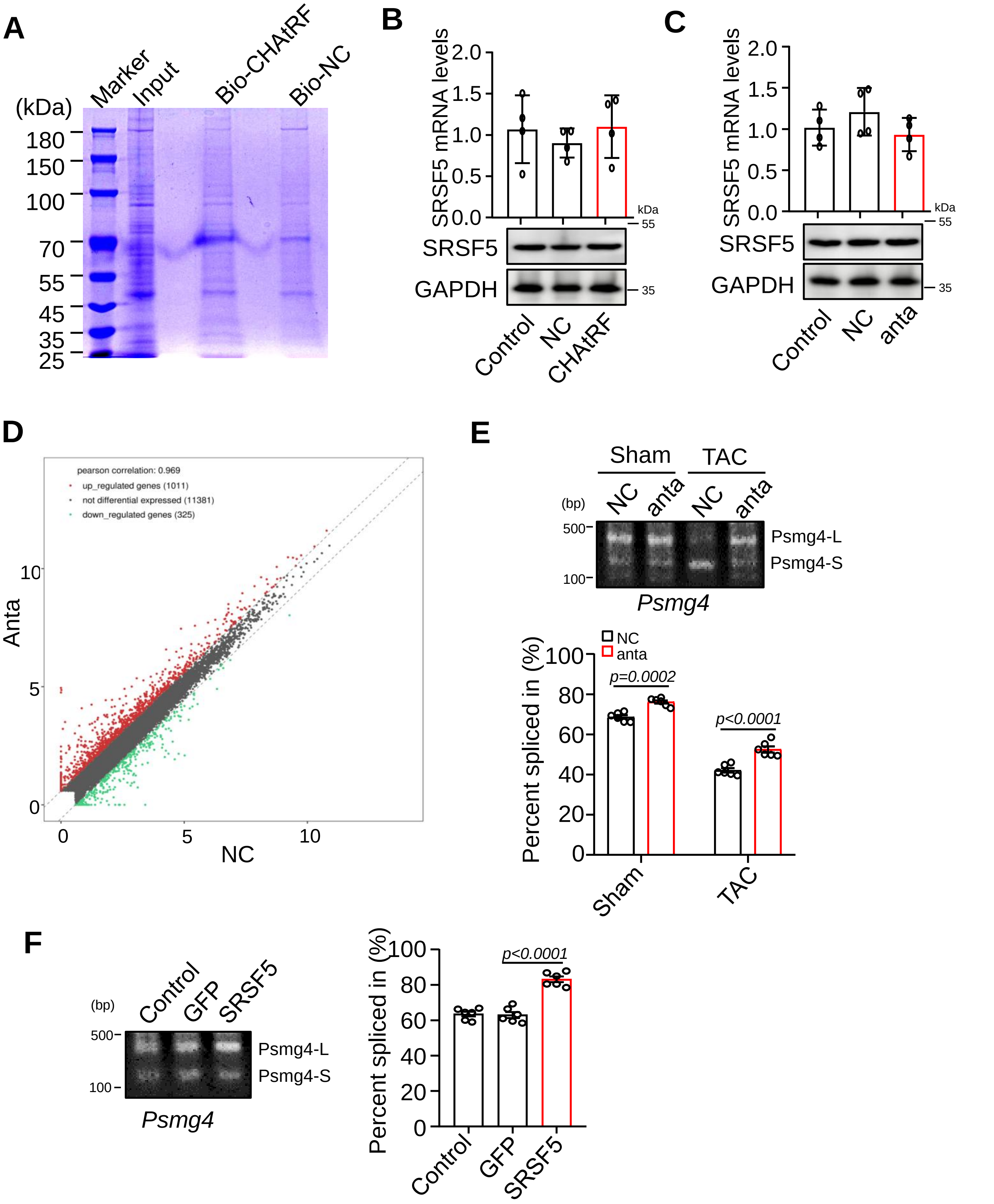

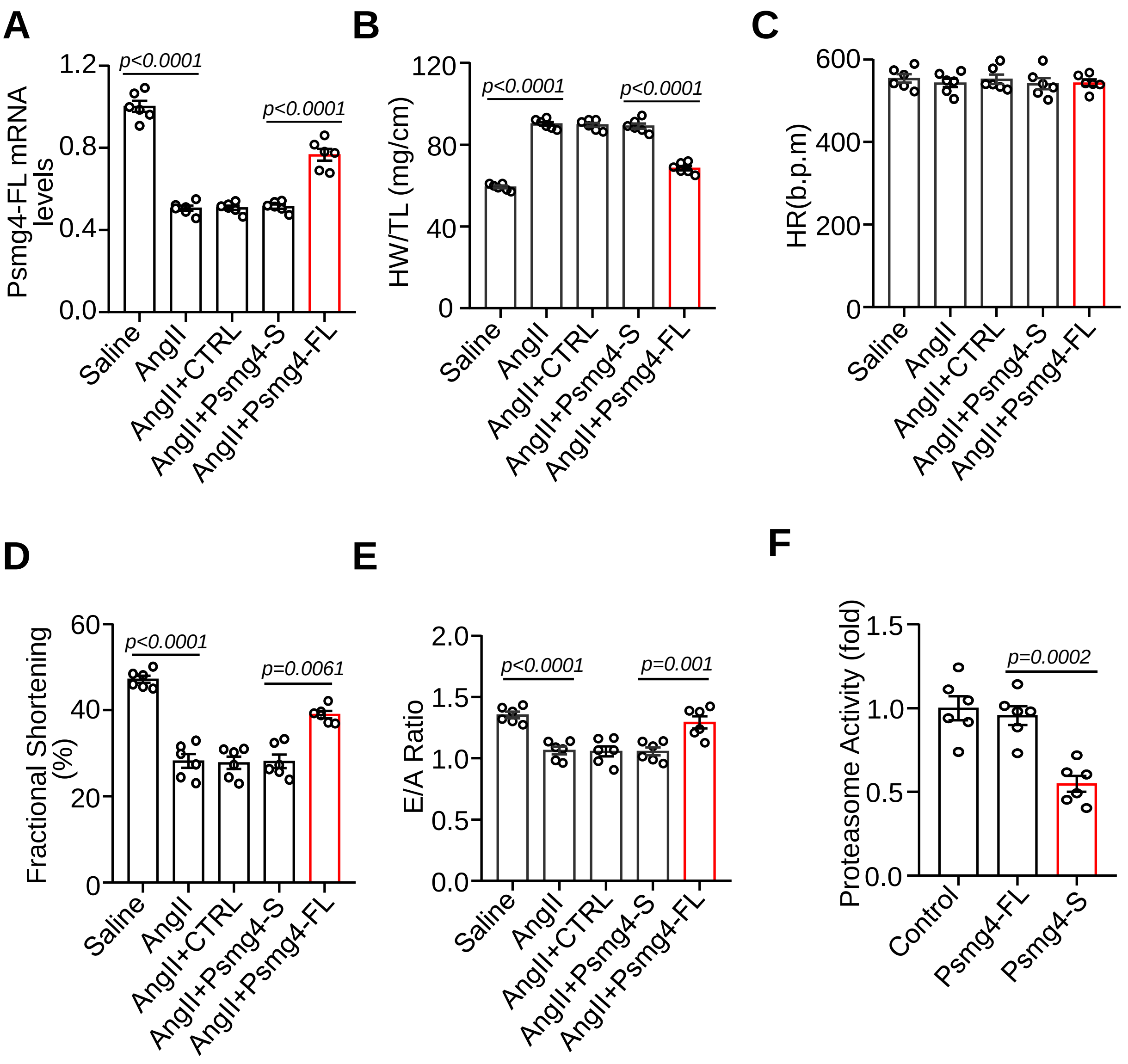
A
Psmg4-FL mRNA levels
p<0.0001
p<0.0001
1.2
0.8
0.4
0.0
Saline
AngII
AngII+CTRL
AngII+Psmg4-S
AngII+Psmg4-FL
B
HW/TL (mg/cm)
p<0.0001
p<0.0001
120
80
40
0
C
HR(b.p.m)
600
400
200
0
D
Fractional Shortening (%)
p<0.0001
p=0.0061
60
40
20
0
E
E/A Ratio
p<0.0001
p=0.001
2.0
1.5
1.0
0.5
0.0
F
Proteasome Activity (fold)
p=0.0002
1.5
1.0
0.5
0.0
Control
Psmg4-FL
Psmg4-S

Supplementary Figure 6

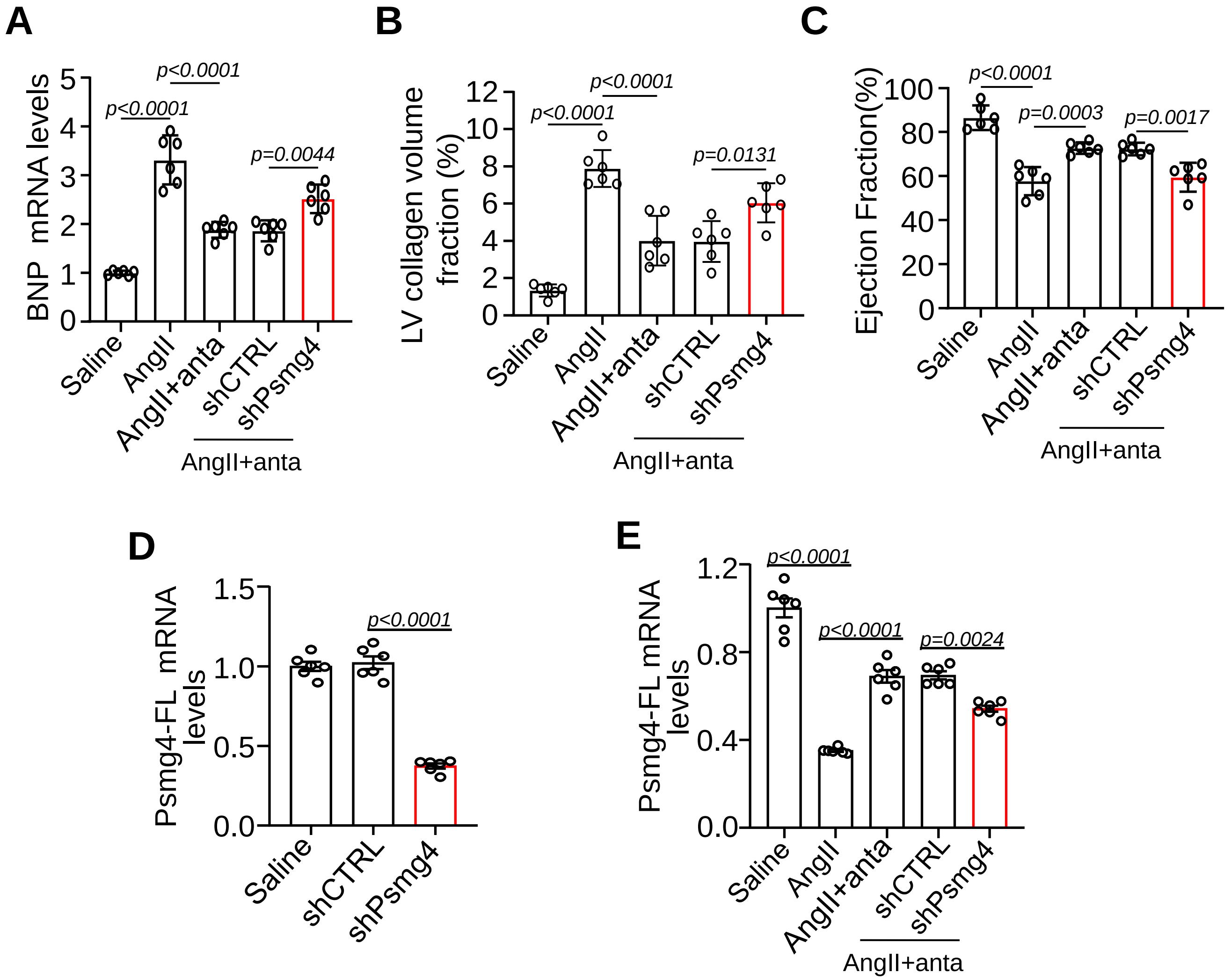

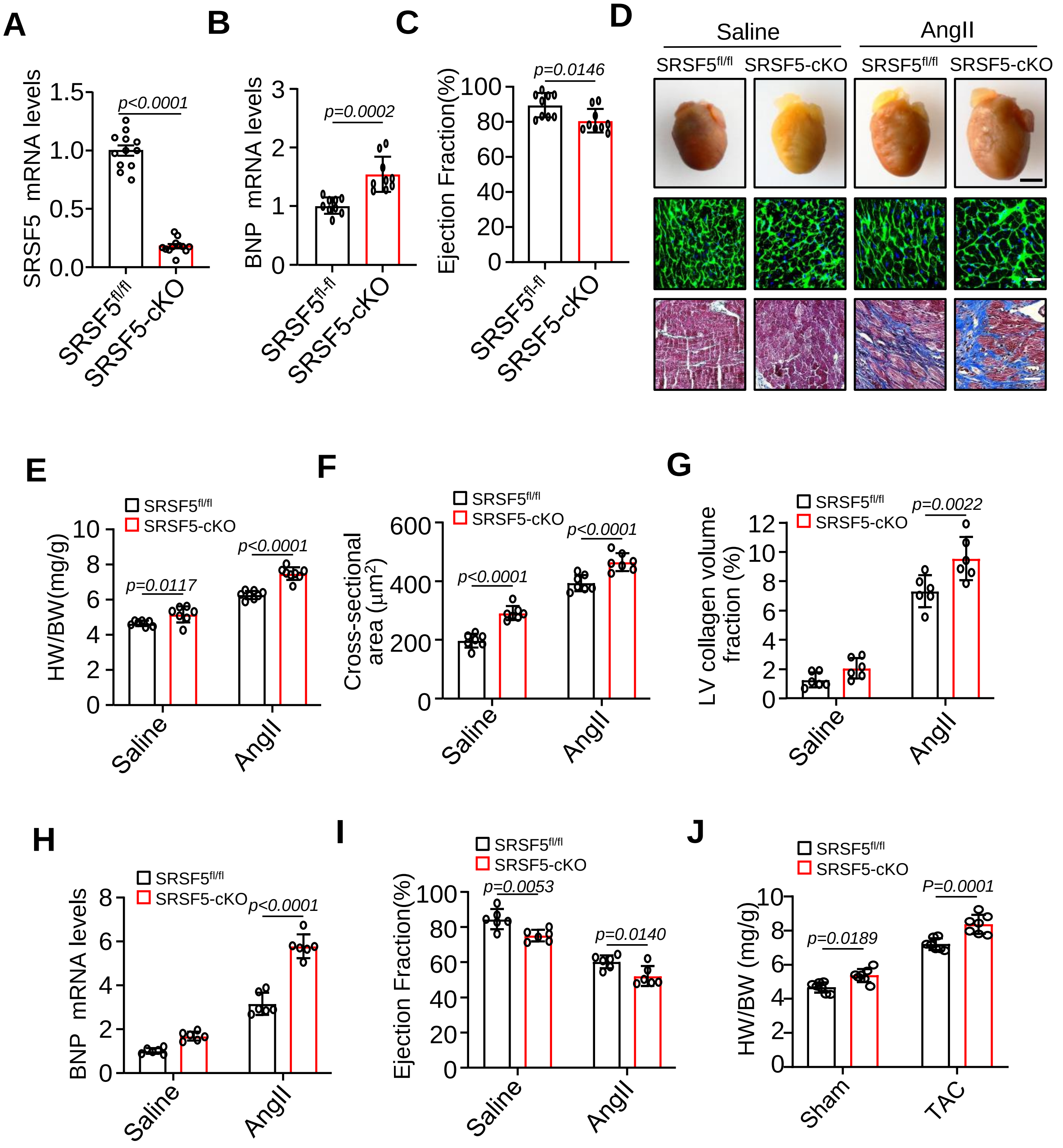
A
B
C
D
Saline
AngII
SRSF5fl/fl
SRSF5-cKO
SRSF5 mRNA levels
p<0.0001
BNP mRNA levels
p=0.0002
Ejection Fraction(%)
p=0.0146
SRSF5fl-fl
E
F
G
HW/BW(mg/g)
p=0.0117
p<0.0001
Cross-sectional area (μm2)
LV collagen volume fraction (%)
p=0.0022
H
I
J
p=0.0053
p=0.0140
HW/BW (mg/g)
p=0.0189
P=0.0001
Sham
TAC

Supplementary Figure 8

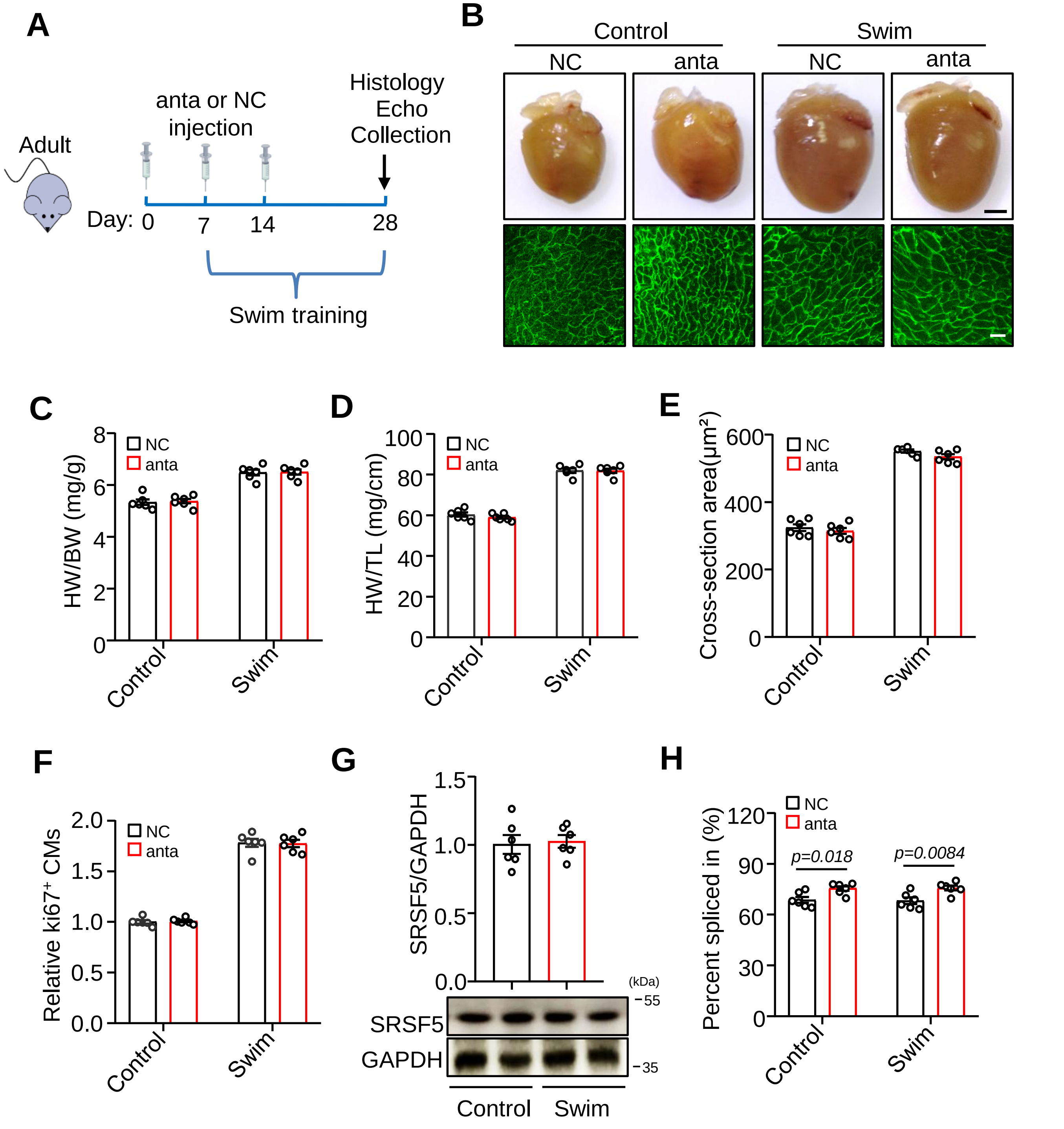

Supplementary Figure 9

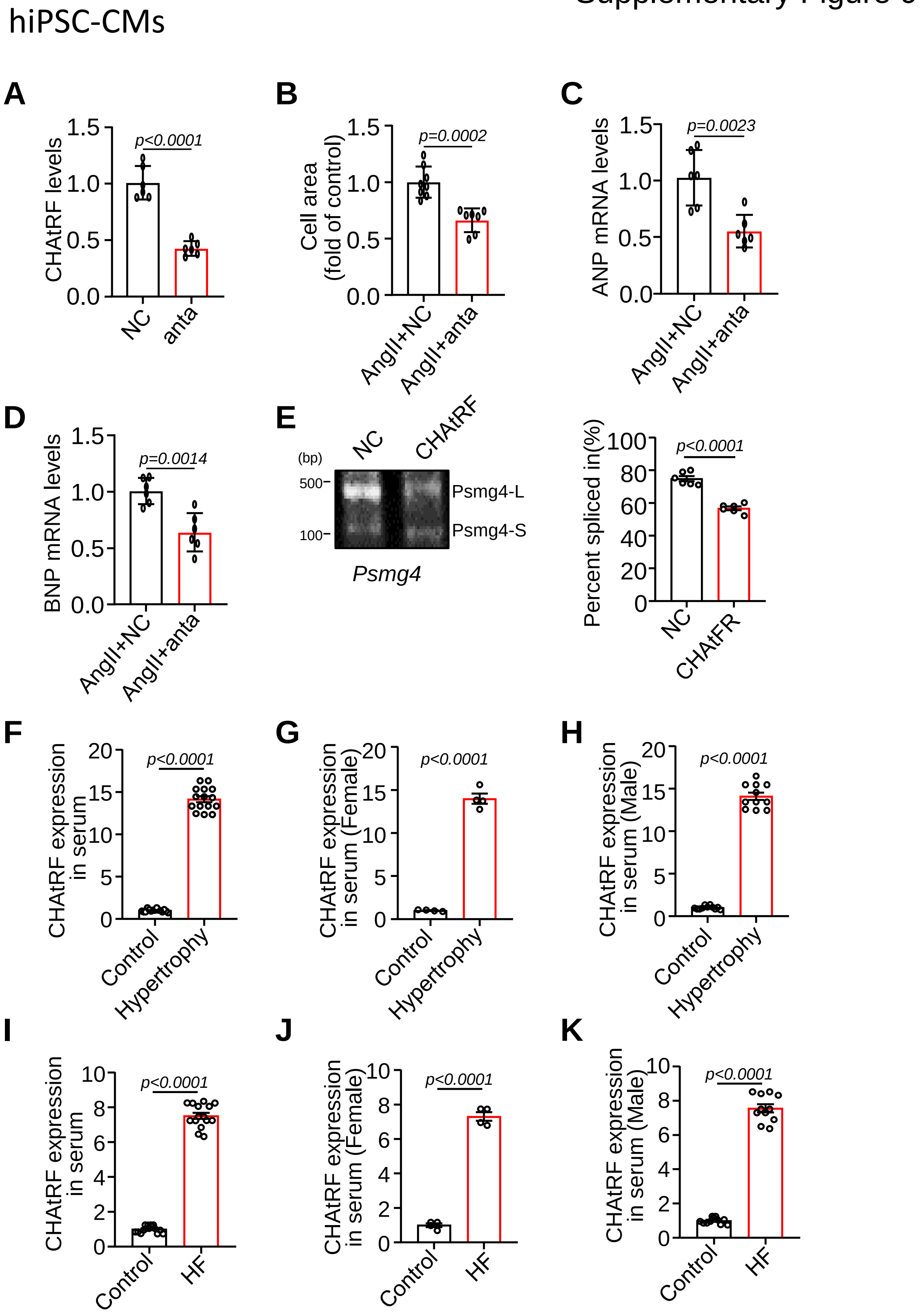

Supplement: Supplementary 1 — Figs. S1 to S9 Tables S1 to S7 [file research.1202.f1.zip › Supplementary materials.pdf]
