# Supplementary material for: CHAtRF Modulates Cardiac Hypertrophy via SRSF5-Dependent Regulation of Psmg4 Alternative Splicing
Source: Research (Wash D C). 2026 Mar 26;9:1202. doi: 10.34133/research.1202 (PMC13018653; doi:10.34133/research.1202)
Supplement: Supplementary 1 — Figs. S1 to S9 Tables S1 to S7 [file research.1202.f1.zip › Table S2.doc]

**Table S2. Clinical characteristics of patients with HCM in Figure 1D.**

Patient 1

| **Parameters** | **Index** |
| --- | --- |
| **Age(y)** | 55 |
| **Sex** | Male |
| **Baseline cardiac disease** | HCM |
| **Hypertension** | N |
| **Diabetes** | N |
| **Ejection Fraction (Simpson%)** | 73 |
| **LVID;d (mm)** | 39 |
| **Ventricular septal thickness;d(mm)** | 19 |
| **Pressure difference of left ventricular outflow tract(mmHg)** | 39 |
| **SAM** | Yes |

Patient 2

| **Parameters** | **Index** |
| --- | --- |
| **Age(y)** | 52 |
| **Sex** | Female |
| **Baseline cardiac disease** | HCM |
| **Hypertension** | N |
| **Diabetes** | N |
| **Ejection Fraction (Simpson%)** | 64 |
| **LVID;d (mm)** | 46 |
| **Ventricular septal thickness;d(mm)** | 18 |
| **Pressure difference of left ventricular outflow tract(mmHg)** | 92 |
| **SAM** | Yes |

Patient 3

| **Parameters** | **Index** |
| --- | --- |
| **Age(y)** | 48 |
| **Sex** | Male |
| **Baseline cardiac disease** | HCM |
| **Hypertension** | Yes |
| **Diabetes** | N |
| **Ejection Fraction (Simpson%)** | 60 |
| **LVID;d (mm)** | 48 |
| **Ventricular septal thickness;d(mm)** | 21 |
| **Pressure difference of left ventricular outflow tract(mmHg)** | 41 |
| **SAM** | Yes |

Patient 4

| **Parameters** | **Index** |
| --- | --- |
| **Age(y)** | 53 |
| **Sex** | Female |
| **Baseline cardiac disease** | HCM |
| **Hypertension** | N |
| **Diabetes** | N |
| **Ejection Fraction (Simpson%)** | 56 |
| **LVID;d (mm)** | 41 |
| **Ventricular septal thickness;d(mm)** | 23 |
| **Pressure difference of left ventricular outflow tract(mmHg)** | 114 |
| **SAM** | Yes |

Patient 5

| **Parameters** | **Index** |
| --- | --- |
| **Age(y)** | 47 |
| **Sex** | Male |
| **Baseline cardiac disease** | HCM |
| **Hypertension** | N |
| **Diabetes** | N |
| **Ejection Fraction (Simpson%)** | 60 |
| **LVID;d (mm)** | 40 |
| **Ventricular septal thickness;d(mm)** | 27 |
| **Pressure difference of left ventricular outflow tract(mmHg)** | 56 |
| **SAM** | Yes |

**Abbreviations**

| **tsRNAs** | TRNA-derived small RNAs |
| --- | --- |
| **AngⅡ** | Angiotensin Ⅱ |
| **tRFs** | tRNA-derived fragments |
| **CHAtRF** | Cardiac hypertrophy associated tRF |
| **HF** | Heart failure |
| **CVDs** | Cardiovascular diseases |
| **SRSF5** | Serine/arginine-rich splicing factor 5 |
| **PSMG4** | Proteasome assembly chaperone 4 |
| **AS** | Alternative splicing |
| **cKO** | Conditional knockout |
| **TAC** | Transverse aortic constriction |
| **HCM** | Hypertrophic cardiomyopathy |
| **anta** | Antagomir |
| **hiPSC-CMs** | Human induced pluripotent stem cell-induced cardiomyocytes |
